# Oral administration of ellagic acid mitigates perioperative neurocognitive disorders, hippocampal oxidative stress, and neuroinflammation in aged mice by restoring IGF-1 signaling

**DOI:** 10.1038/s41598-024-53127-8

**Published:** 2024-01-30

**Authors:** Fang Chen, Kai Lu, Ning Bai, Yabo Hao, Hui Wang, Xinrong Zhao, Fang Yue

**Affiliations:** Department of Anesthesiology, Shaanxi Provincial People’s Hospital, The Third Affiliated Hospital of Xi’an Jiaotong University, Xi’an Jiaotong University, Xi’an, 710068 Shaanxi China

**Keywords:** Cognitive ageing, Cognitive neuroscience, Learning and memory

## Abstract

This study investigates the potential of ellagic acid (EA), a phytochemical with antioxidant and anti-inflammatory properties, in managing perioperative neurocognitive disorders (PND). PND, which represents a spectrum of cognitive impairments often faced by elderly patients, is principally linked to surgical and anesthesia procedures, and heavily impacted by oxidative stress in the hippocampus and microglia-induced neuroinflammation. Employing an aged mice model subjected to abdominal surgery, we delve into EA's ability to counteract postoperative oxidative stress and cerebral inflammation by engaging the Insulin-like growth factor-1 (IGF-1) pathway. Our findings revealed that administering EA orally notably alleviated post-surgical cognitive decline in older mice, a fact that was manifested in improved performance during maze tests. This enhancement in the behavioral performance of the EA-treated mice corresponded with the rejuvenation of IGF-1 signaling, a decrease in oxidative stress markers in the hippocampus (like MDA and carbonylated protein), and an increase in the activity of antioxidant enzymes such as SOD and CAT. Alongside these, we observed a decrease in microglia-driven neuroinflammation in the hippocampus, thus underscoring the antioxidant and anti-inflammatory roles of EA. Interestingly, when EA was given in conjunction with an IGF1R inhibitor, these benefits were annulled, accentuating the pivotal role that the IGF-1 pathway plays in the neuroprotective potential of EA. Hence, EA could serve as a potent candidate for safeguarding against PND in older patients by curbing oxidative stress and neuroinflammation through the activation of the IGF-1 pathway.

## Introduction

Perioperative neurocognitive disorders (PND) represent a spectrum of cognitive abnormalities that that manifest after surgical procedures and the administration of anesthesia. These can present as postoperative disorientation, postponed cognitive recuperation, or persistent cognitive deterioration^1^. It is noted that approximately one in five patients encounter these neurocognitive deficits within the initial three months post-surgery, affecting broad cognitive domains including behavior, mood, memory, attention, and judgement abilities. The resulting cognitive changes can have substantial repercussions on daily life, social exchanges, and self-reliance^1^.

PND is commonly associated with advanced age, as almost 40% of elderly individuals experience symptoms of postoperative delirium or cognitive impairment^2^. Intriguingly, oxidative stress, a cardinal feature of cellular aging, tends to amplify within the brain during the aging process^3,4^. This amplification could be attributed to a gradual diminution in conventional antioxidant defenses such as superoxide dismutase (SOD)^5^, in concert with declining mitochondrial functionality^6^. This heightened vulnerability may lead to damage to proteins, lipids, and DNA, thereby proposing a plausible path towards neurological disorders such as Alzheimer's Disease (AD)^7,8^ and Parkinson's disease (PD)^9,10^. Moreover, heightened post-surgery oxidative stress in the hippocampus has been noted in elderly animal models^11,12^, a condition likely exacerbated by neuroinflammation mediated by microglia^13^. Surgical stress appears to prime hippocampal microglial activation^14,15^, which can be particularly accentuated in elderly subjects^14^. Consequently, approaches focused on antioxidative and anti-inflammatory interventions have been identified as a promising strategy for managing PNDs in the elderly population.

In this context, insulin-like growth factor-1 (IGF-1), a neurotrophic hormone critical for brain development and maturation^16,17^, merits our attention. IGF-1 significantly influences cellular neuroplasticity via its specific receptor IGF1R, playing a crucial role in learning and memory processes^18^. It has demonstrated protective capabilities in a range of brain disorders, including AD^19^, PD^20^, stroke^21^, by modulating neuronal survival, differentiation, synaptogenesis, and synaptic plasticity. One mechanism through which IGF-1 imparts its neuroprotective effects involves bolstering the antioxidative response^22^. It can enhance the levels of antioxidative enzymes such as SOD and catalase (CAT), and decrease levels of ROS and lipid peroxidation products^22,23^. Additionally, IGF-1 can regulate microglia-mediated neuroinflammation^16,24^. It can inhibit microglial activation and lessen the generation of inflammation-promoting agents by suppressing the NF-κB pathway in microglia^16,25^. IGF-1 also possesses the capability to stimulate the production of anti-inflammatory substances, including interleukin-10 (IL-10), fostering microglial polarization to the anti-inflammatory M2 state^24,25^. Notably, earlier clinical studies have implied a potential correlation between decreased plasma IGF-1 levels and the onset of PND^26^. Collectively, these pieces of evidence suggest that IGF-1 may represent a vital target for countering oxidative damage and microglia-induced neuroinflammation in PND.

The therapeutic potential of plant-derived natural products in neuroprotection is well-documented^27,28^. Our previous research highlighted the efficacy of β-caryophyllene in alleviating PND via activation of hippocampal CB2 receptors^29^. Building on this, we turned our attention to ellagic acid (EA), a polyphenolic compound abundant in nuts, vegetables, and fruits^30^. EA is renowned for its antioxidative and anti-inflammatory properties, crucial in combating neuroinflammation and oxidative damage^30,31^. Its neuroprotective effects have been demonstrated in models of traumatic brain injury and degenerative neurological disorders^32–34^. Crucially, experimental evidence lends support to the neuroprotective effects of EA as demonstrated in animal models of traumatic brain injury^32^ and degenerative neurological disorders^33,34^. These studies collectively indicate that EA may hold a key role in curtailing cognitive deterioration and tempering cerebral damage, primarily through the suppression of neuroinflammation and oxidative damage. However, it remains uncertain whether EA treatment could assuage PND in the aged by stimulating hippocampal IGF-1 signaling.

Hence, the main objective of our research is to explore the potential of employing EA as a plant-based substitute treatment for PND in aged mice. We posit that EA may alleviate oxidative stress and neuroinflammation in aged mice, specifically via the initiation of the IGF-1 pathway.

## Materials and methods

### Animals

This study adhered to the ethical guidelines outlined in the National Institutes of Health (NIH) publication, “Care and Use of Laboratory Animals” (NIH Publications No. 8023, updated in 1978). The research subjects consisted of 18-month-old male C57BL/6J mice, weighing between 26 and 33 g, obtained from the Laboratory Animal Center at Xi’an Jiaotong University. This study, encompassing all experiments involving animals, was carried out in line with the ARRIVE guidelines. Moreover, all experimental procedures received the requisite approval from the Animal Care and Use Committees of Xi’an Jiaotong University. Prior to commencing the experiment, the mice were provided unrestricted access to standard mouse feed and water.

### Establishment of the experimental model of PND in aged mice

In our effort to establish a preclinical model of Perioperative Neurocognitive Disorders (PND) in elderly mice, we adopted an approach involving abdominal surgery under pentobarbital anesthesia. This methodology, referenced from a previous study^35^ and corroborated by our recent work^29^, was chosen due to the high prevalence of abdominal surgery in clinical settings and its recognized utility in research exploring perioperative cognitive dysfunction^36,37^. After the abdominal area was disinfected and shaved, a 2-cm midline incision was made. Subsequently, gentle manipulation of both the small intestine and colon was performed for approximately 20 s, repeating the procedure within a span of 20 min. To minimize differences in surgical technique, a single surgeon performed all operations. For the sham procedure, the mice underwent disinfection and received pentobarbital sodium anesthesia, but no abdominal surgery was performed. After surgery, postoperative pain was controlled by injecting 1% ropivacaine (100 μL) at the surgical site.

### Pharmacological intervention and group allocation

The aged mice were orally administered ellagic acid (EA) at either 50 or 100 mg/kg (Cat# E808704, Macklin, Shanghai, China) via gavage (200 μL, dissolved in 10% DMSO in saline) for a period of seven consecutive days preceding the surgical procedure. The purpose was to assess the impact of EA on hippocampal IGF-1 signaling post-surgery. The mice were divided into four cohorts: (1) Sham group, where they were given an oral vehicle solution (200 μL of 10% DMSO in saline) daily for a week followed by a sham operation; (2) Surgery group, where the vehicle solution was provided daily for a week before an abdominal surgery to initiate the PND model; (3) EA 50 mg/kg group, where they received oral EA at a rate of 50 mg/kg each day for a week before abdominal surgery to create the PND model; (4) EA 100 mg/kg group, where they were given oral EA at a rate of 100 mg/kg daily for a week before abdominal surgery. The selection of the EA dosage was grounded in prior research^38^, which demonstrated the efficacy of these dosages in the treatment of neurological disorders through oral administration.

The ensuing phase of our investigation was geared toward assessing the influence of EA on postoperative cognitive performance, hippocampal oxidative stress, and neuroinflammation. Moreover, to probe the interaction between EA and IGF-1 signaling, we administered an intraperitoneal injection of the IGF1R inhibitor picropodophyllin (PPP) (Cat# 407247, Merck Millipore, Billerica, MA, USA) 30 min prior to treatment with EA. A solution of PPP was formulated by dissolving it in a mixture of 0.1% DMSO in saline. Mice were allocated to one of four cohorts: (1) Sham group, which received both oral and intraperitoneal vehicle solutions daily (200 μL and 100 μL respectively) for a week, culminating in a Sham procedure; (2) Surgery group, which was provided with the same volumes of oral and intraperitoneal vehicle solutions each day for a week, preceding an abdominal surgery to instigate the PND model; (3) EA group, which was given daily oral EA at a dose of 100 mg/kg and a 100 μL intraperitoneal vehicle solution for a week, ending with abdominal surgery; (4) EA + PPP group, where daily oral EA of 100 mg/kg was combined with an intraperitoneal injection of PPP at 1 mg/kg for a week before abdominal surgery. The regimen for PPP treatment was established based on prior research^39^, which has demonstrated its efficacy in Alzheimer’s Disease transgenic mice.

### Behavioral tests

Five days post-surgery, the mice were subjected to the Open Field (OPF) test to assess their locomotor activity and exploratory capability. Metrics taken into consideration included the distance traversed in the center zone and the time duration spent in this zone. Each mouse underwent a single testing session in this setup. Each mouse was initially positioned in a black square open-top box, measuring 40 cm × 40 cm × 40 cm, with its nose pointed towards the wall in a corner. A 20 cm × 20 cm central area of the box was marked as the central zone, with the remainder serving as the peripheral zone. A camera positioned directly over the box monitored the mouse’s free exploration for a span of 5 min. In order to prevent any carryover effects, the base of the box was carefully cleaned with 75% ethanol to eliminate any residual traces left by the preceding mouse before each new testing session commenced.

The Morris Water Maze (MWM) apparatus consists of a circular pool measuring 120 cm in diameter and 60 cm in depth, filled with water up to a height of 45 cm. A platform with a diameter of 10 cm is positioned 1.5 cm below the surface of the water. The water in the pool is meticulously maintained at a steady temperature of 21 ± 1 °C. Any external cues remained unchanged throughout the experiment.

Five days post-surgery, the mice were subjected to daily training trials for a consecutive five-day period. Each day incorporated three training periods, interspersed with five-minute breaks. The duration taken to discover the platform (termed escape latency) was documented. The average of escape latency derived from the three daily trials was employed for statistical computations. In instances where a mouse did not succeed in finding the platform within a span of 75 s, it was gently steered towards the platform, recording the latency as 75 s.

Three hours subsequent to the last training trial, a probe test was executed with the platform being eliminated. The trajectory of the mouse over a span of 90 s was monitored, concentrating the analysis on the duration expended in the target quadrant and the frequency of crossing the former platform’s location.

### Western blotting and ELISA-based protein quantification

The method for extracting protein from mouse hippocampus (n = 6/group) was referenced from our recently published article^29^. The harvested protein supernatant was carefully extracted and split evenly for subsequent analyses via Western blot and ELISA methods.

ELISA assays were conducted using commercial IL-10 and IL-6 kits (Cat# PI522 for IL-10, Cat# PI326 for IL-6, Beyotime, Shanghai, China) following the manufacturer’s instructions. Western blot analysis involved separating the samples (40 μg per well) using 12% SDS-PAGE gel electrophoresis. The proteins were subsequently transferred to a PVDF membrane and blocked with 5% skim milk or 3% bovine serum albumin for two hours at room temperature. After careful washing with TBST, the membranes were exposed to specific antibodies such as anti- Arginase-1 (Arg-1) (1:1000; Cat# A1847, Abclonal, Wuhan, China), anti-IGF-1 (1:500; Cat# ab9572, Abcam, Cambridge, UK), anti-p-IGF1R (1:500; Cat# AP0367, Abclonal, Wuhan, China), anti- inducible nitric oxide synthase (iNOS) (1:1000; Cat# A14031, Abclonal, Wuhan, China), anti-total IGF1R (1:1000; Cat# ab182408, Abcam), and anti-β-actin (1:4000; Cat# AC006, Abclonal) overnight at 4 °C. After additional TBST washes, HRP-conjugated IgG (1:5000; Beyotime) was applied to the membranes for one hour at room temperature. The proteins were then detected using ECL (Cat# P0018FS, Beyotime) and the grayscale values were recorded and statistically processed with ImageLab software. The protein level of p-IGF1R was normalized to total IGF1R, and the calculations for normalization and average values were based on data from three independent experiments done in triplicate.

### Assessment of oxidative damage

We used an additional six mice from each group to assess the levels of oxidative stress-related indicators and proteins associated with the nuclear factor erythroid 2-related factor 2 (Nrf2) signaling pathway. After extracting the hippocampal tissues from the mice, we fully lysed and homogenized the samples, then collected the supernatant and measured protein concentration using BCA assays (as illustrated in above Western blot section). Half of the tissue supernatants were utilized for assessing oxidative stress-related indicators, while the other half were employed for Western blot analysis to detect the protein levels of nuclear Nrf2 and HO-1.

In order to assess oxidative stress damage, we quantified the levels of oxidative stress markers—Malondialdehyde (MDA) and carbonyl protein—and antioxidant enzymes, specifically Superoxide Dismutase (SOD) and Catalase (CAT). All these markers were evaluated using commercially available assay kits (MDA kit, Cat# S0131, Beyotime; carbonyl protein kit, Cat# ab126287, Abcam; SOD kit, Cat# S0101, Beyotime; CAT kit, Cat# S0051, Beyotime), stringently following the protocols supplied by the respective manufacturers.

Nrf2 is a critical transcription factor that modulates the antioxidant proteins and a multitude of genes that confer protection against oxidative damage triggered by inflammation and injury^40^. Upon the onset of oxidative stress, Nrf2 migrates to the nucleus and orchestrates the transcription of specific downstream genes, including heme oxygenase-1 (HO-1)^41^. Extraction of the nuclear protein fraction from hippocampal tissue was achieved by utilizing a Nuclear and Cytoplasmic Protein Extraction Kit (Cat# S0131, Beyotime), strictly conforming to the protocol provided by the manufacturer. The quantification of Nrf2 and HO-1 in the nuclear fraction was carried out through Western blotting, using the aforementioned protocol with primary antibodies such as anti-HO-1 (1:500; Cat# A1346, Abclonal, Wuhan, China) and anti-Nrf2 (1:1000; Cat# A0674, Abclonal). The nuclear protein levels of Nrf2 and HO-1 were subsequently normalized to LaminB, as detected by rabbit anti-LaminB (1:1000, Cat# A1910, Abclonal).

### Quantitative polymerase chain reaction

RNA was extracted from hippocampal tissue via the Trizol technique (Invitrogen Life Technologies), which was then followed by cDNA reverse transcription facilitated by a kit from Takara (Tokyo, Japan). The subsequent quantitative real-time polymerase chain reaction (qRT-PCR) made it possible to ascertain the ΔCt value during the period of exponential amplification. The PCR reaction process was as follows: an initial pre-denaturation phase at 94 °C for 15 min, denaturation at 94 °C for a span of 10 s, and annealing/extension at 66 °C for 30 s, repeating this sequence for 40 cycles. Each sample underwent testing in triplicate using the ΔΔCt method for assessing shifts in gene expression, with adjustments made relative to GAPDH.

The primers implemented in the study included: GAPDH (forward 5′-ACTCCACTCACGGCAAATTC-3′, reverse 5′-TCTCCATGGTGGTGAAGACA-3′), IGF-1 (forward 5′-TGCCCTCAACCCCACTACTG-3′, reverse 5′-CGGTTGTCACTGGTTCATGTG-3′), CD86 (forward 5′-CATGGGCTTGGCAATCCTTA-3′, reverse 5′-AAATGGGCACGGCAGATATG-3′), iNOS (forward 5′-ACATCGACCCGTCCACAGTAT-3′, reverse 5′-CAGAGGGGTAGGCTTGTCTC-3′), CD206 (forward 5′-CTCTGTTCAGCTATTGGACGC-3′, reverse 5′-TGGCACTCCCAAACATAATTTGA-3′), and Arg-1 (forward 5′-CTCCAAGCCAAAGTCCTTAGAG-3′, reverse 5′-GGAGCTGTCATTAGGGACATCA-3′).

### Immunofluorescence analysis

For immunofluorescence analysis, mice were perfused with a solution containing 4% formaldehyde. Subsequently, the tissues were embedded in paraffin to generate 5-μm thick sections. The sections underwent antigen retrieval in a Citrate Buffer solution (pH 6.0) in a microwave oven. Afterward, the sections were exposed to antibodies that specifically targeted Iba-1 (Cell Signaling Technology, Beverly, MA, Cat# 17198, 1:400). The secondary antibody incubation employed DyLight® 488-conjugated goat anti-rabbit antibody (Abcam, Cat# ab96899), diluted at 1:200. Furthermore, nuclear staining was performed using DAPI.

To visualize the hippocampal dentate gyrus (DG) and CA1 regions, sections were examined under an Olympus microscope (Tokyo, Japan) equipped with a 40 × objective lens. The captured images were analyzed for Iba-1-positive cell count and immunoreactivity using Image Pro™ Plus software.

### Statistical analysis

All outcomes are presented as the mean ± Standard Deviation (SD). Data from MWM tests, specifically escape latency, were examined statistically using Two-way ANOVA. To compare platform crossing times, the Kruskal–Wallis ANOVA method was employed. Other data sets were evaluated using one-way ANOVA, subsequently followed by Dunnett’s multiple comparisons test. All statistical procedures were carried out utilizing GraphPad Prism 5 software, developed by GraphPad Software Inc., USA. A p-value below 0.05 was considered to indicate statistical significance.

## Results

### Ellagic acid (EA) administration counters surgical disruption of IGF-1 signaling in the hippocampus of aged mice

**Figure 1:**
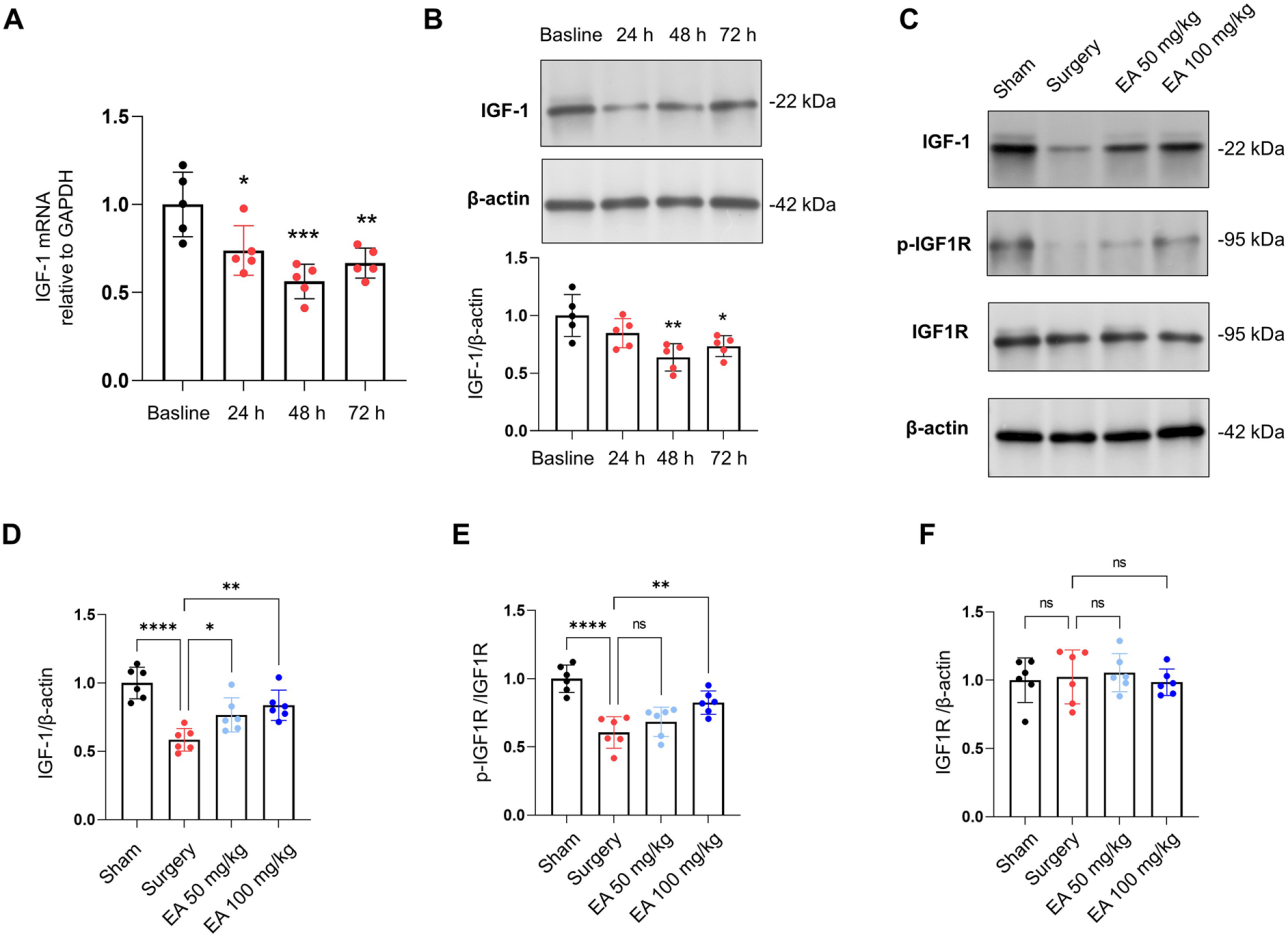
EA treatment mitigates the surgical-induced suppression of IGF-1 signaling. (**A**) Temporal variations in hippocampal IGF-1 mRNA expression after surgery were determined using qPCR, observing changes at Baseline, and 24, 48, and 72 h post-surgery. (**B**) Hippocampal IGF-1 protein expression was evaluated via western blot at baseline and at 24, 48, and 72 h following surgery. (**C**–**F**) The impact of oral EA treatment on hippocampal IGF-1 and p-IGF1R levels in aged mice was assessed. The protein levels of IGF-1 and IGF1R were normalized to β-actin, and the levels of p-IGF1R were normalized to total IGF1R. The data are presented as mean ± SD. Statistical significance is indicated as *P < 0.05, **P < 0.01, ***P < 0.001, and ****P < 0.0001, while 'ns' represents non-significant results.

Prior clinical research has indicated a potential link between postoperative cognitive dysfunction and diminished levels of IGF-1 in the blood plasma^26^. However, the specific influence of surgical interventions on hippocampal IGF-1 protein expression in animal models remains unclear. Our study aimed to explore the changes in hippocampal IGF-1 protein expression after surgery in aged mice. Our findings revealed a decrease in hippocampal IGF-1 mRNA and protein expression within 24 h following surgery, bottoming out at various intervals within the subsequent 48 h, before gradually increasing again (Fig. 1A,B, *n* = 5; for IGF-1 mRNA, *P* = 0.0173 at 24 h, *P* = 0.0003 at 48 h, *P* = 0.0030 at 72 h; for IGF-1 protein, *P* = 0.2092 at 24 h, *P* = 0.0016 at 48 h, *P* = 0.0164 at 72 h). The 48-h mark, where the lowest hippocampal IGF-1 expression was observed, was chosen for further experimentation.

In order to evaluate EA's impact on PND, we orally administered EA to aged mice at either 50 mg/kg or 100 mg/kg, once daily for a week before surgery. The data showed that all EA-treated mice, regardless of dose, displayed a notable increase in hippocampal IGF-1 protein levels than the Surgery group (Fig. 1C,D, *n* = 6, *P* = 0.0249 for 50 mg/kg EA, *P* = 0.0021 for 100 mg/kg EA). Given the positive correlation between IGF-1 signaling activity and phosphorylated IGF1R (p-IGF1R)^42^, we assessed the hippocampal level of p-IGF1R. The outcomes mirrored those of IGF-1: the Surgery group had significantly lower hippocampal p-IGF1R levels compared to the Sham group (Fig. 1C,E, *n* = 6, *P* < 0.0001), while mice receiving 100 mg/kg EA had significantly higher levels than the Surgery group (Fig. 1C,E, *n* = 6, *P* = 0.4329 for 50 mg/kg EA, *P* = 0.0041 for 100 mg/kg EA). Nonetheless, no substantial disparities in the total protein levels of IGF1R were detected among the different experimental groups. (Fig. 1C,F, *n* = 6,* F* (3, 20) = 0.2368, *P* = 0.8696).

### Enhancement of IGF-1 signaling through Oral EA administration mitigates postoperative cognitive impairment in aged mice

**Figure 2:**
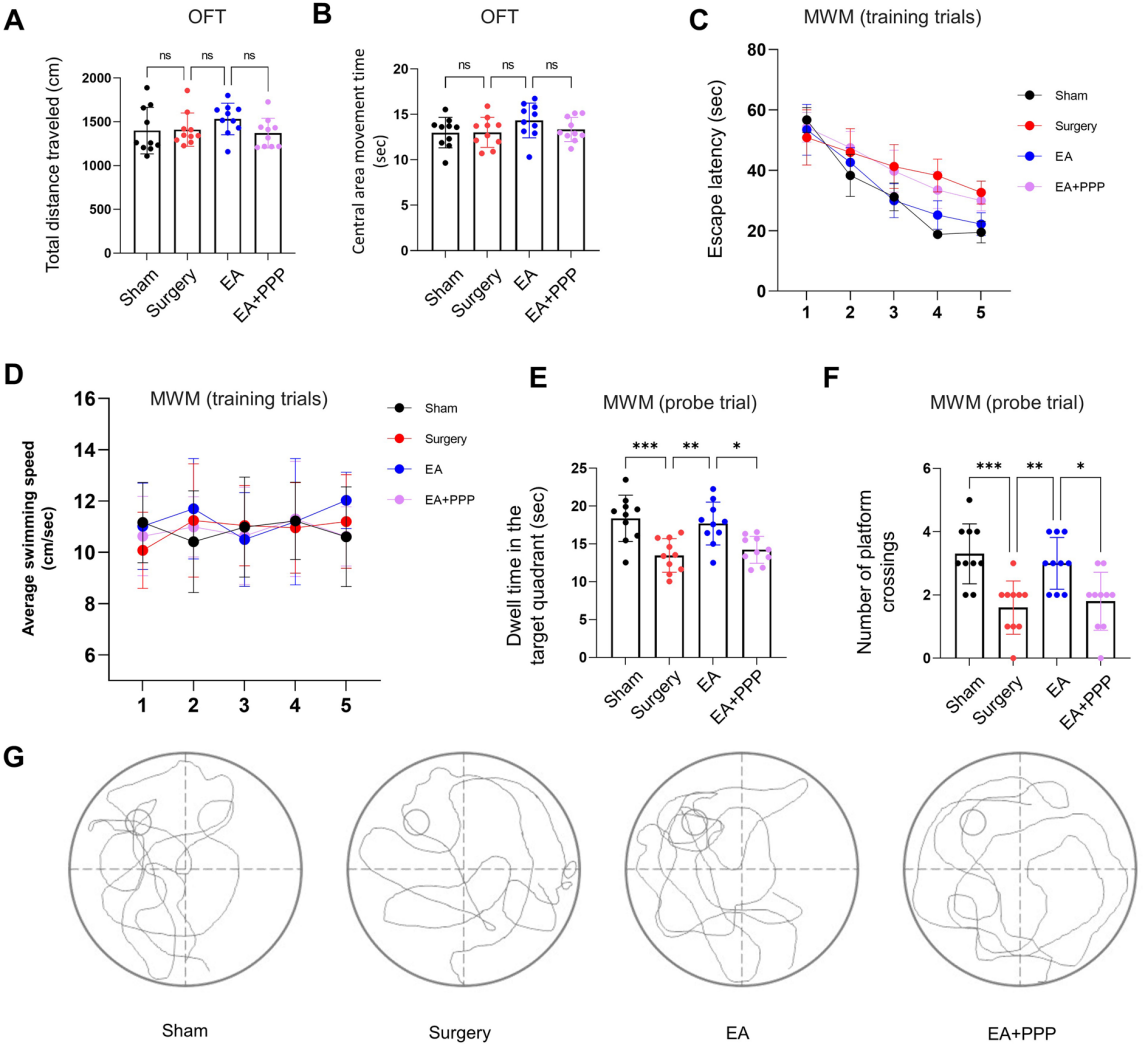
Oral administration of EA effectively mitigated postoperative cognitive dysfunction by activating the IGF-1 signaling pathway. (**A**) The total distance traveled in the OFT on postoperative day 5 was measured. Prior to abdominal surgery, the EA group were administered a daily oral dosage of 100 mg/kg EA for a week. In contrast, the EA + PPP group received intraperitoneal injections of the IGF1R antagonist PPP at a dosage of 10 mg/kg, 30 min prior to each EA administration. (**B**) The time spent in the central area during the OFT on postoperative day 5 was recorded. (**C**) The escape latency during Training trials in the Morris Water Maze (MWM) experiment, conducted between postoperative days 6 and 10, was evaluated. (**D**) The average swimming speed during the Training trials in the MWM experiment was also measured. Two hours after the last Training trial, during the Probe trials in the MWM experiment with the platform removed, the time spent in the target quadrant and the number of crossings at the original platform location within 75 s were recorded. These results are presented as (**E**) dwell time in the target quadrant and (**F**) number of platform crossings during the Probe trial. (**G**) Exemplary movement tracks of mice from each group during the Probe trial of the MWM experiment are depicted. The data are presented as mean ± SD. Statistical significance is denoted as *P < 0.05, **P < 0.01, and ***P < 0.0001, while 'ns' indicates non-significant results.

In order to assess the impact of EA on postoperative cognitive function, we employed the OFT and the MWM test. To minimize the potential confounding influence of surgery or sham operations on the animals' motor ability, the OFT was conducted on the fifth postoperative day, and the MWM test was administered from the sixth to the tenth postoperative day. OFT results did not reveal significant differences in total movement distance and time spent in the central area among the Sham, Surgery, and EA groups (which received oral EA 100 mg/kg) (Fig. 2A,B, *n* = 10, for total movement distance, *P* = 0.3164,* F* (3, 36) = 1.220; for central area movement time, *P* = 0.2492,* F* (3, 36) = 1.433).

Results from the MWM test, as illustrated in Fig. 2C–G, demonstrated that mice in the Surgery group exhibited a significantly prolonged escape latency compared to the Sham group (training trials, *n* = 10, two-way ANOVA, *P* < 0.0001,* F* (1, 18) = 97.54), with no notable alteration in average moving speed (training trials, *n* = 10, two-way ANOVA, *P* = 0.9322,* F* (1, 18) = 0.0075). Additionally, the Surgery group demonstrated a decrease in target quadrant dwell time (*n* = 10, *P* = 0.0003) and reduced platform crossing instances (*n* = 10, *P* = 0.0007) during the probe trial. In contrast to the Surgery group, the EA group of aged mice exhibited a significant decrease in escape latency during the training trials (*n* = 10, two-way ANOVA, *P* < 0.0001, *F* (1, 18) = 45.54). Moreover, during the probe trial, these mice demonstrated notable increases in both the dwell time in the target quadrant and the number of platform crossings (n = 10, *P* = 0.0035 for target quadrant dwell time and *P* = 0.0059 for platform crossing instances). These results suggest that oral delivery of EA (100 mg/kg) can alleviate cognitive dysfunction stemming from abdominal surgery in aged mice.

To determine whether EA ameliorated postoperative cognitive performance in aged mice by rejuvenating the IGF-1 signaling pathway, we administered PPP, an IGF1R antagonist^43^, intraperitoneally at a dosage of 10 mg/kg^44^, 30 min before each EA treatment (EA + PPP group). Relative to the EA group, the EA + PPP group demonstrated a marked elevation in escape latency (Fig. 2C, training trials, *n* = 10, two-way ANOVA, *P* < 0.0001, *F* (1, 18) = 26.29), a pronounced reduction in time spent in the target quadrant (Fig. 2E, probe trial, *n* = 10, *P* = 0.0204), and a reduced number of platform crossings (Fig. 2F, probe trial, *n* = 10, *P* = 0.0220). However, no significant variations related to PPP co-administration were found in terms of average swimming speed (Fig. 2D, training trials, *n* = 10, two-way ANOVA, *P* = 0.2245,* F* (1, 18) = 1.583), total distance traveled (Fig. 2A, n = 10, *P* = 0.3043), or central area movement time during the Open Field Test (Fig. 2B, n = 10, *P* = 0.5423).

### EA activates the IGF-1 signaling pathway to counter postoperative hippocampal oxidative stress damage in aged mice

**Figure 3:**
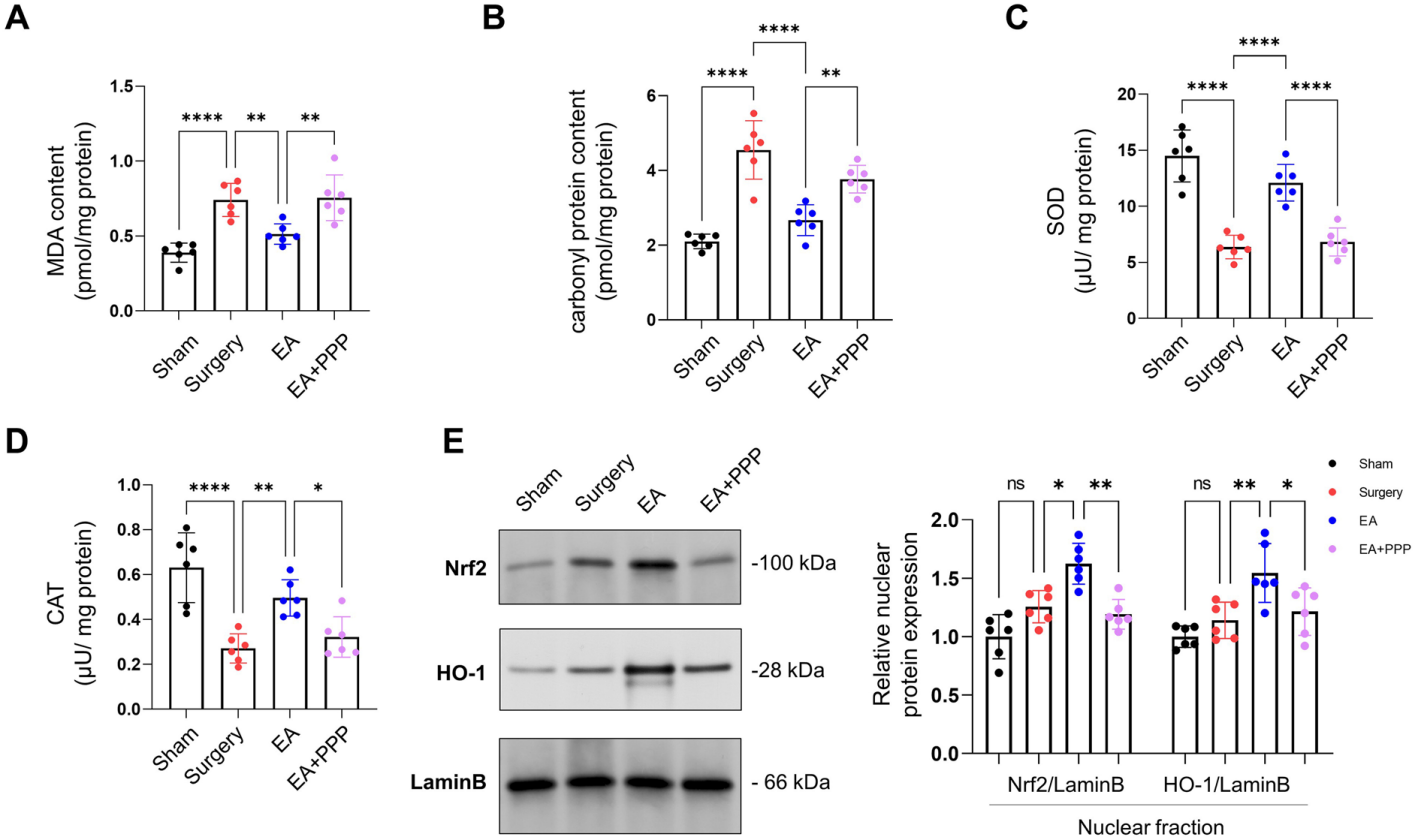
EA administration orally mitigated postoperative hippocampal oxidative stress damage in aged mice through the IGF-1 signaling pathway. (**A**) Hippocampal MDA levels were measured 48 h post-surgery. (**B**) Hippocampal carbonyl protein content (indicative of protein oxidation) was assessed at the same 48-h mark. (**C**) SOD activity in the hippocampus was determined 48 h following surgery. (**D**) CAT activity in the hippocampus was evaluated 48 h post-surgery. (**E**) Protein levels of nuclear Nrf2 and HO-1 in the hippocampus were quantified 48 h after the surgical procedure. Data are represented as mean ± SD. Statistical significance is denoted as *P < 0.05, **P < 0.01, ***P < 0.001, and ****P < 0.0001, while 'ns' indicates non-significance.

We then evaluated EA’s impact on oxidative stress in the hippocampus. The surgery group showed elevated levels of oxidative stress markers (MDA and carbonylated protein) and reduced antioxidant enzyme activities (SOD and CAT). Our findings showed a significant escalation in hippocampal MDA and carbonyl protein content in the Surgery group, 48 h post-surgery, in contrast to the Sham group (Fig. 3A–D, *n* = 6, *P* < 0.0001 for MDA, *P* < 0.0001 for carbonyl protein content). Concurrently, the Surgery group exhibited decreased SOD and CAT levels (Fig. 3A–D, *n* = 6, *P* < 0.0001 for SOD, *P* < 0.0001 for CAT). However, the EA group showed significant reductions in MDA and carbonyl protein content, alongside increased SOD and CAT levels (Fig. 3A–D, *n* = 6, *P* = 0.0061 for MDA, *P* < 0.0001 for carbonyl protein content, *P* < 0.0001 for SOD, *P* = 0.0061 for CAT). Notably, the antioxidant response of EA was found to be diminished by the IGF1R inhibitor PPP, marked by elevated MDA and carbonyl protein content and reduced SOD and CAT levels (Fig. 3A–D, *n* = 6, *P* = 0.0036 for MDA, *P* = 0.0047 for carbonyl protein content, *P* < 0.0001 for SOD, *P* = 0.0390 for CAT).

Additionally, our findings indicated that, relative to the sham group, mice receiving only the abdominal surgery displayed a slight increase in nuclear Nrf2 and HO-1, although these variations did not attain statistical significance (Fig. 3E, n = 6, *P* = 0.1965 for Nrf2, *P* = 0.8486 for HO-1). Furthermore, oral administration of EA boosted the protein amount of nuclear Nrf2 and HO-1 in mice that underwent surgical procedures (Fig. 3E, n = 6, *P* = 0.0138 for Nrf2, *P* = 0.0049 for HO-1). However, this effect was nullified upon the introduction of the IGF1R inhibitor PPP (Fig. 3E, n = 6, *P* = 0.0022 for Nrf2, *P* = 0.0375 for HO-1).

### EA treatment mitigates postoperative activation of hippocampal microglia in aged mice via the IGF-1 signaling pathway

**Figure 4:**
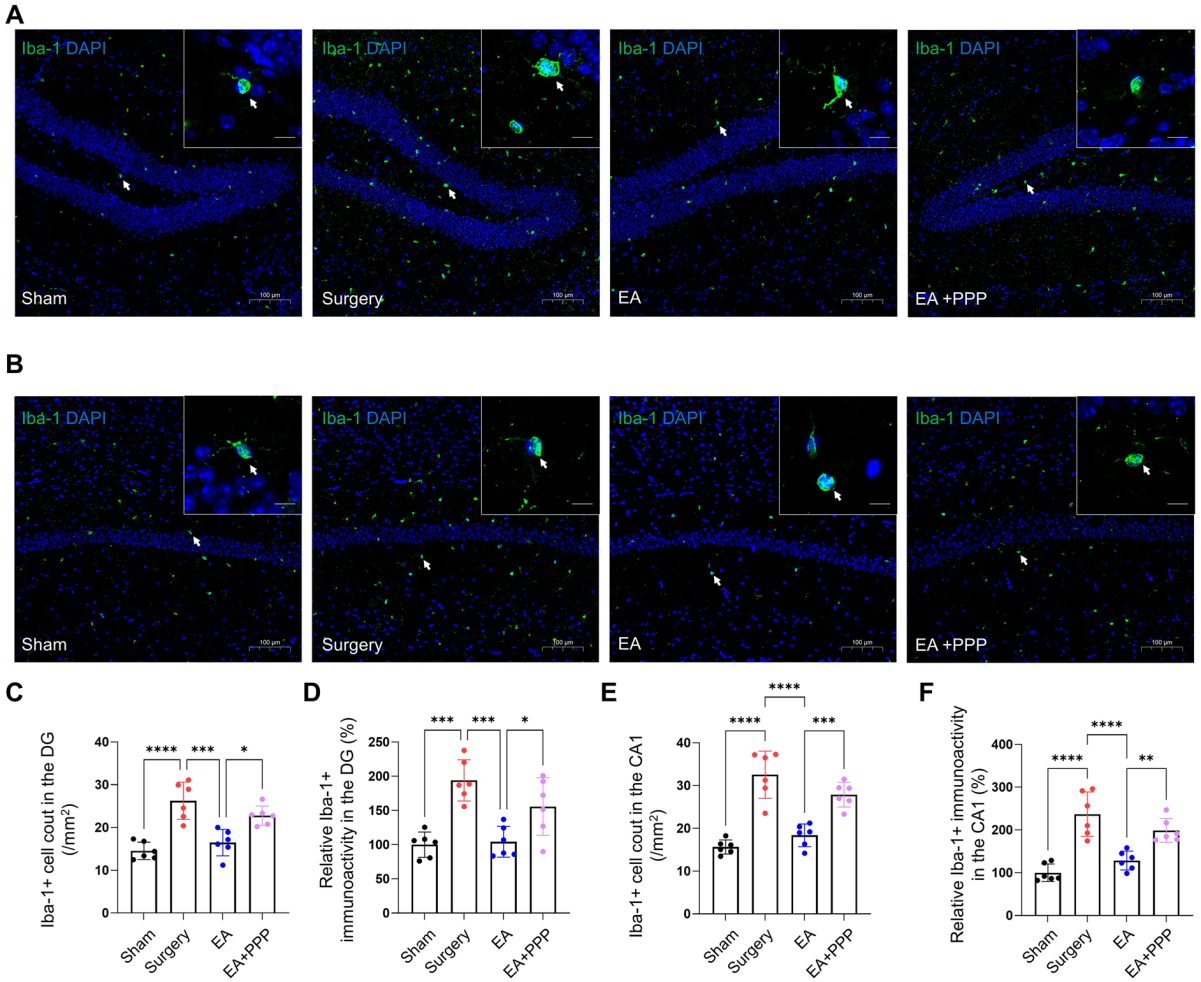
The inhibitory effect of orally administered EA on the postoperative activation of hippocampal microglia in aged mice is mediated by the IGF-1 signaling. (**A**) Iba-1 immunofluorescence staining (green for Iba-1, blue for DAPI) of the hippocampal CA1 region in aged mice post EA oral administration is displayed, with a 100 μm scale bar for reference. A representative Iba-1 + cell, indicated by a white arrow, is magnified in the upper right inset (scale bar 10 μm). (**B**) The same staining of the hippocampal DG region in aged mice following EA oral administration is shown. A magnified view of a representative Iba-1 + cell, pointed out with a white arrow, is provided in the upper right inset (scale bar 10 μm).The results of Iba-1 positive microglial cell count in the CA1 and DG regions are portrayed in (**C**) and (**E**), respectively, whereas (**D**) and (**F**) depict the results of Iba-1 immunofluorescence intensity in these corresponding regions. Data are presented as mean ± SD. Statistical significance is indicated by the symbols *P < 0.05, **P < 0.01, ***P < 0.001, and ****P < 0.0001, while the term 'ns' denotes non-significance.

Considering the crucial role of microglia in hippocampal postoperative neuroinflammation^45^, we explored the effects of EA on microglia in the hippocampal CA1 and DG regions. Our data revealed noteworthy decreases in both the quantity of Iba-1-positive microglia and the immunoactivity of Iba-1 in the DG and CA1 regions of mice orally administered with EA, observed 48 h after the surgical procedure (Fig. 4A–F, *n* = 6, *P* = 0.0001 for Iba-1 + cell count in DG, *P* = 0.0002 for Iba-1 + fluorescence intensity in DG, *P* < 0.0001 for Iba-1 + cell count in CA1, *P* < 0.0001 for Iba-1 + fluorescence intensity in CA1). However, PPP was able to mitigate EA’s suppressive effect on microglial activation, as indicated by an increase in quantity of Iba-1-positive microglia and the immunoactivity of Iba-1 in the CA1 and DG regions (Fig. 4A–F, *n* = 6, *P* = 0.0103 for Iba-1 + cell count in DG, *P* = 0.0332 for Iba-1 + fluorescence intensity in DG, *P* = 0.0007 for Iba-1 + cell count in CA1, *P* = 0.0072 for Iba-1 + fluorescence intensity in CA1).

### The IGF-1 signaling pathway modulates the shift of microglial activation states from M1 to M2 following EA administration

Given the pivotal role of IGF-1 in the regulation of microglial polarization, as highlighted in several studies^46,47^, especially in M1/M2 polarization—where M1 and M2 represent pro-inflammatory and anti-inflammatory microglial activation states respectively—we proceeded to evaluate the M1 and M2 microglial markers. Additionally, we analyzed the cytokines IL-6, associated with M1 activation, and IL-10, which is linked with M2 activation, within the hippocampal region.

**Figure 5:**
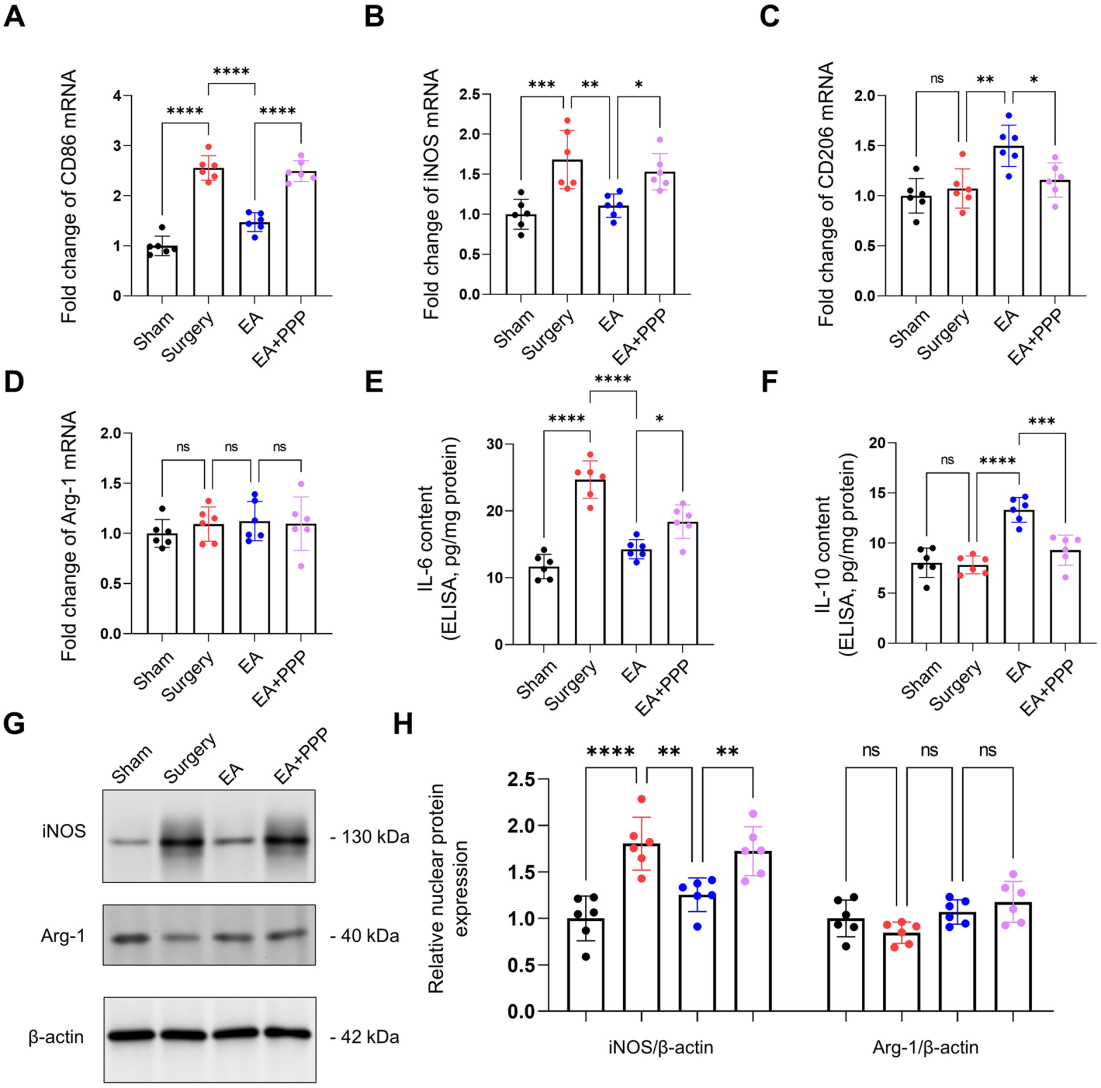
EA administration via oral route shifts microglial activation states from M1 to M2 through the IGF-1 signaling pathway. The mRNA fold change of M1 markers CD86 and iNOS are represented in panels (**A**) and (**B**), respectively. Panels (**C**) and (**D**) display the levels of M2 markers CD206 and Arg-1 in the hippocampus. The concentrations of the cytokines IL-6 and IL-10, as determined by ELISA, are shown in panels (**E**) and (**F**) respectively. The immunoreactivity of GFAP did not show significant changes among the four experimental groups. The data are presented as mean ± SD. Statistical significance is denoted by *P < 0.05, **P < 0.01, ***P < 0.001, and ****P < 0.0001, while 'ns' indicates no significant difference.

Our experimental results reveal a considerable augmentation in the mRNA expression levels of the M1 microglial markers, namely CD86 and iNOS, as well as the protein level of iNOS within the Surgery group, relative to the Sham group (Fig. 5A,B,D, *n* = 6, *P* < 0.0001 for CD86 mRNA, *P* = 0.0006 for iNOS mRNA, *P* < 0.0001 for iNOS protein). Conversely, the mRNA expression levels of the M2 markers, namely CD206 and Arg-1, in conjunction with the protein level of Arg-1, remained unchanged, displaying no statistically significant variations (Fig. 5C,D, *n* = 6, *P* = 0.9045 for CD206 mRNA, *P* = 0.8482 for Arg-1 mRNA, *P* = 0.9036 for Arg-1 protein).

Administration of EA led to decreased mRNA levels of CD86 and iNOS and increased CD206 mRNA level, with no impact on Arg-1 mRNA (Fig. 5A–D, *n* = 6, *P* < 0.0001 for CD86, *P* = 0.0031 for iNOS, *P* = 0.0041 for CD206, *P* = 0.9934 for Arg-1). In relation to protein expression, iNOS exhibited a statistically significant rise following EA treatment. In contrast, the protein level of Arg-1 remained stable, showcasing no discernible effect from the EA administration (Fig. 5G,H, *n* = 6, *P* = 0.0014 for iNOS, *P* = 0.6021 for Arg-1). An interesting observation was made in the co-administration scenario involving EA and PPP. The results indicated a mitigation of the effects previously induced by EA. This was made evident by the noted increase in the mRNA levels of CD86 and iNOS (Fig. 5A–D, *n* = 6, *P* < 0.0001 for CD86 mRNA, *P* = 0.0328 for iNOS mRNA), alongside the protein level of iNOS (Fig. 5G,H, *n* = 6, *P* = 0.0095 for iNOS protein, *P* = 0.9856 for Arg-1).

Moreover, our findings showed increased hippocampal IL-6 levels after surgery (Fig. 5E, n = 6, *P* < 0.0001), whereas changes in IL-10 were not statistically significant (Fig. 5F, n = 6, *P* = 0.9924). EA administration notably reduced IL-6 levels and increased IL-10 levels (Fig. 5E, F, *n* = 6, *P* < 0.0001 for IL-6, *P* < 0.0001 for IL-10). Notably, PPP co-administration curbed the effects of EA on hippocampal IL-6 and IL-10 production (Fig. 5E,F, *n* = 6, *P* = 0.0203 for IL-6, *P* = 0.0002 for IL-10).

## Discussion

Our research contributes to the growing body of knowledge on the neuroprotective potential of EA, especially its capacity to mitigate postoperative cognitive impairment in aged mice. The improved behavioral performance observed in mice treated with EA coincided with a recovery of IGF-1 signaling, a decrease in hippocampal oxidative stress by-products, such as MDA and carbonylated protein, along with an increase in antioxidative enzymes, specifically SOD and CAT. Additionally, we observed diminished microglia-related neuroinflammation in the hippocampus, collectively underscoring EA's antioxidative and anti-inflammatory attributes. Our data further pointed to the pivotal role of IGF-1 signaling in mediating EA's neuroprotective effect, as evidenced by the abrogation of these protective effects when an IGF1R inhibitor was co-administered.

Nutritional supplements enriched with EA, such as Ellagic Active tablets produced by Source Naturals (Santa Cruz, CA, USA), have garnered considerable usage due to their purported health-promoting properties^48^. In line with these findings, our approach involved oral pre-treatment of mice with EA prior to undergoing abdominal surgical procedures. Our observations indicate that EA exerts a notable neuroprotective effect on mice experiencing PND. These results imply that a dietary approach, including supplementation with EA-rich foods such as certain nuts and vegetables, could potentially reduce the risk of PND among elderly individuals. Nonetheless, while our study has centered on a murine model, further research involving human clinical trials will be essential to validate the potential efficacy of EA in alleviating PND in elderly human populations.

However, it is pertinent to highlight the absence of an established Recommended Dietary Allowance (RDA) for ellagic acid in human consumption, a factor that complicates the direct translation of our dosages to a human equivalent. Existing studies suggest that the typical daily intake of ellagic acid from natural dietary sources, such as fruits, ranges from approximately 20–50 mg^49^. Consequently, the dosages employed in our study likely surpass what would be ordinarily consumed through a regular diet. Nonetheless, this discrepancy underscores a potential avenue for dietary modifications in humans. Increasing the consumption of fruits and vegetables could feasibly elevate ellagic acid intake to levels that offer neuroprotective benefits, as indicated by our findings.

The antioxidative properties of EA are typically cited as the basis for its neuroprotective potential^30^. Consistent with this, our findings demonstrated that EA not only reduced postoperative hippocampal oxidative stress by-products, including MDA and carbonylated protein, but also increased the activity of antioxidative SOD and CAT. This was further corroborated by the observed enhancement of anti-oxidative Nrf2 signaling activation in the hippocampi of EA-treated mice, as indicated by the elevated levels of nuclear Nrf2 and HO-1.

Our results propose that the stimulation of the IGF-1 signaling pathway could play a central role in facilitating the antioxidative effects attributed to EA. This is particularly evident given our finding that the EA-induced antioxidative effect in the hippocampus following surgery was negated by the co-administration of the IGF1R inhibitor, PPP. Furthermore, a decline in the activity of brain IGF-1 signaling has been noted in correlation with the aging process^17^. Our study revealed that surgical interventions further compromised hippocampal IGF-1 signaling in aged mice, as evidenced by reduced IGF-1 production and decreased levels of phosphorylated IGF1R. Nevertheless, these alterations were rectified in EA-treated mice, implying an enhancing effect of EA on hippocampal IGF-1 signaling. Upon activation, IGF-1 signaling can mitigate oxidative damage by alleviating mitochondrial dysfunction^50^ and triggering downstream antioxidative signals such as Nrf2^51^ and PI3K/Akt^50^ pathways.

In addition to oxidative stress, the microglia-mediated neuroinflammatory response is another fundamental mechanism driving the onset and progression of PND^11,12,45^. Our investigation also revealed a notable surge in the count of microglial cells (Iba-1 +), the intensity of Iba-1 immunoreactivity, and the production of the pro-inflammatory mediator, IL-6, in the hippocampus of aged mice. However, the neuroinflammatory response was attenuated in mice pre-treated with EA, reflecting EA's widely recognized anti-inflammatory properties in diverse neurological conditions, including AD^52^, PD^53^, and cuprizone-induced neurotoxicity^54^.

Microglial activation could be broadly classified into M1 (classical) and M2 (alternative) phenotypes^55,56^. The detrimental M1 phenotype releases pro-inflammatory cytokines and reactive oxygen species, whereas the beneficial M2 phenotype is associated with tissue repair and resolution of inflammation^55,56^. Relating this to PND, it is worth noting that aged mice display decreased M2 polarization and intensified M1 polarization compared to normal adults^57^. Corroborating this, we observed post-surgical increases in M1 markers (CD86 and iNOS) mRNA levels in the aged mice's hippocampus, without significant shifts in M2 markers (CD206 and Arg-1) mRNA levels or the anti-inflammatory cytokine IL-10. Remarkably, mice treated with EA demonstrated a decrease in M1 markers coupled with an increase in the M2 marker CD206, hinting at EA's potential to catalyze a transition in hippocampal microglia from a pro-inflammatory M1 to an anti-inflammatory M2 phenotype. This corresponds with recent studies showcasing the ability of oral EA treatment to bolster M2 microglial polarization in the brains of rats subjected to LPS treatment^58^.

Moreover, our data suggest that the anti-inflammatory benefits of EA treatment are closely tied to its activation of the IGF-1 signaling pathway. We observed that EA's reduction of microglial proliferation and its enhancement of M2 markers were impeded by co-administration of the IGF1R inhibitor PPP. IGF-1 and its receptor IGF1R are found in a wide array of cell types in the central nervous system, including neurons^59,60^, astrocytes^60,61^, and macrophage/microglia^60,62^. IGF-1 is recognized for its anti-inflammatory role in the brain, which it accomplishes by managing microglial inflammation^63^, the reduction of pro-inflammatory factors^24,64^, and modulation of astrocytic phagocytosis^65^. Moreover, the activation of IGF-1 signaling promotes a shift towards the anti-inflammatory M2 phenotype in microglia following cerebral hemorrhage^64^. Therefore, in light of these findings, we propose that EA's neuroprotective effects against postoperative hippocampal neuroinflammation are at least partially mediated through its stimulation of IGF-1 signaling.

While our study yields promising results, it is important to consider its limitations. A primary limitation pertains to the interaction between EA treatment and the modulation of IGF-1 signaling. The complexity of the IGF-1 pathway, which is influenced by a multitude of factors such as growth hormones^66^, oxidative stress^66^, and microRNAs^67^, poses a significant challenge. Although existing research, including studies on Caenorhabditis elegans, suggests that EA may enhance stress resistance through the insulin/IGF-1 pathway^68^, the broad biological effects of EA, encompassing antioxidative and anti-inflammatory properties, could impact IGF-1 signaling in various ways. Consequently, there is a compelling need for further investigation into the specific mechanisms of EA's interaction with IGF-1 signaling. Moreover, our study, while emphasizing EA's antioxidative and anti-inflammatory effects through IGF-1 signaling modulation, does not preclude the possibility that EA might enhance perioperative cognitive function through other molecular signaling pathways, such as microRNAs. Additionally, our interpretation of M1/M2 microglial phenotypes warrants caution. The binary classification of microglia into M1 and M2 simplifies a complex process. Recent studies suggest a spectrum of microglial activation states, challenging this dichotomy^69^. Our findings, indicating EA's influence on microglial polarization, contribute to this narrative but might not fully capture microglia's nuanced roles in neuroinflammation. Future research should focus on characterizing microglia's diverse activation states in response to EA and understanding their role in neuroinflammatory processes related to PND.

In conclusion, our study provides compelling evidence that EA holds significant neuroprotective potential against postoperative cognitive impairment in aged mice. This protection is demonstrated through improvements in behavioral performance, reductions in oxidative stress, and the attenuation of neuroinflammation in the hippocampus of EA-treated mice. Key to these effects is EA's ability to stimulate IGF-1 signaling, which appears to mediate its antioxidative and anti-inflammatory impacts. Furthermore, EA's accessibility in dietary form presents an appealing avenue for further research into dietary interventions to mitigate the risk of PND in elderly individuals (Supplementary Information S1).

### Supplementary Information

Supplementary Figures.

## Data Availability

The datasets used and/or analysed during the current study available from the corresponding author on reasonable request.

## References

[1] Kong H, Xu LM, Wang DX (2022). Perioperative neurocognitive disorders: A narrative review focusing on diagnosis, prevention, and treatment. CNS Neurosci. Ther..

[2] Eckenhoff RG, Maze M, Xie Z, Culley DJ, Goodlin SJ, Zuo Z, Wei H, Whittington RA, Terrando N, Orser BA, Eckenhoff MF (2020). Perioperative neurocognitive disorder: State of the preclinical science. Anesthesiology.

[3] Junqueira VB, Barros SB, Chan SS, Rodrigues L, Giavarotti L, Abud RL, Deucher GP (2004). Aging and oxidative stress. Mol. Aspects Med..

[4] Finkel T, Holbrook NJ (2000). Oxidants, oxidative stress and the biology of ageing. Nature.

[5] de Cavanagh EM, Piotrkowski B, Fraga CG (2004). Concerted action of the renin-angiotensin system, mitochondria, and antioxidant defenses in aging. Mol. Aspects Med..

[6] Sun N, Youle RJ, Finkel T (2016). The mitochondrial basis of aging. Mol. Cell.

[7] Zhang YH, Wang DW, Xu SF, Zhang S, Fan YG, Yang YY, Guo SQ, Wang S, Guo T, Wang ZY, Guo C (2018). α-Lipoic acid improves abnormal behavior by mitigation of oxidative stress, inflammation, ferroptosis, and tauopathy in P301S Tau transgenic mice. Redox. Biol..

[8] McGrath LT, McGleenon BM, Brennan S, McColl D, Mc IS, Passmore AP (2001). Increased oxidative stress in Alzheimer's disease as assessed with 4-hydroxynonenal but not malondialdehyde. QJM.

[9] Hanan M, Simchovitz A, Yayon N, Vaknine S, Cohen-Fultheim R, Karmon M, Madrer N, Rohrlich TM, Maman M, Bennett ER, Greenberg DS, Meshorer E, Levanon EY, Soreq H, Kadener S (2020). A Parkinson's disease CircRNAs resource reveals a link between circSLC8A1 and oxidative stress. EMBO Mol. Med..

[10] Yan MH, Wang X, Zhu X (2013). Mitochondrial defects and oxidative stress in Alzheimer disease and Parkinson disease. Free Rad. Biol. Med..

[11] Netto MB, de Oliveira Junior AN, Goldim M, Mathias K, Fileti ME, da Rosa N, Laurentino AO, de Farias BX, Costa AB, Rezin GT, Fortunato JJ, Giustina AD, Barichello T, Dal-Pizzol F, Petronilho F (2018). Oxidative stress and mitochondrial dysfunction contributes to postoperative cognitive dysfunction in elderly rats. Brain Behav. Immun..

[12] Liu Q, Sun YM, Huang H, Chen C, Wan J, Ma LH, Sun YY, Miao HH, Wu YQ (2021). Sirtuin 3 protects against anesthesia/surgery-induced cognitive decline in aged mice by suppressing hippocampal neuroinflammation. J. Neuroinflam..

[13] Vilhardt F, Haslund-Vinding J, Jaquet V, McBean G (2017). Microglia antioxidant systems and redox signalling. Br. J. Pharmacol..

[14] Zhang ZJ, Zheng XX, Zhang XY, Zhang Y, Huang BY, Luo T (2020). Aging alters Hv1-mediated microglial polarization and enhances neuroinflammation after peripheral surgery. CNS Neurosci. Ther..

[15] Li B, Dasgupta C, Huang L, Meng X, Zhang L (2020). MiRNA-210 induces microglial activation and regulates microglia-mediated neuroinflammation in neonatal hypoxic-ischemic encephalopathy. Cell Mol. Immunol..

[16] Bhalla S, Mehan S, Khan A, Rehman MU (2022). Protective role of IGF-1 and GLP-1 signaling activation in neurological dysfunctions. Neurosci. Biobehav. Rev..

[17] Ashpole NM, Sanders JE, Hodges EL, Yan H, Sonntag WE (2015). Growth hormone, insulin-like growth factor-1 and the aging brain. Exp. Gerontol..

[18] Soto M, Cai W, Konishi M, Kahn CR (2019). Insulin signaling in the hippocampus and amygdala regulates metabolism and neurobehavior. Proc. Natl. Acad. Sci. USA.

[19] Talbot K, Wang HY, Kazi H, Han LY, Bakshi KP, Stucky A, Fuino RL, Kawaguchi KR, Samoyedny AJ, Wilson RS, Arvanitakis Z, Schneider JA, Wolf BA, Bennett DA, Trojanowski JQ, Arnold SE (2012). Demonstrated brain insulin resistance in Alzheimer's disease patients is associated with IGF-1 resistance, IRS-1 dysregulation, and cognitive decline. J. Clin. Invest..

[20] Wang XW, Yuan LJ, Yang Y, Zhang M, Chen WF (2020). IGF-1 inhibits MPTP/MPP(+)-induced autophagy on dopaminergic neurons through the IGF-1R/PI3K-Akt-mTOR pathway and GPER. Am. J. Physiol. Endocrinol. Metab..

[21] Zhang J, Liu M, Huang M, Chen M, Zhang D, Luo L, Ye G, Deng L, Peng Y, Wu X, Liu G, Ye W, Zhang D (2019). Ginsenoside F1 promotes angiogenesis by activating the IGF-1/IGF1R pathway. Pharmacol. Res..

[22] Higashi Y, Sukhanov S, Anwar A, Shai SY, Delafontaine P (2010). IGF-1, oxidative stress and atheroprotection. Trends Endocrinol. Metab..

[23] Puche JE, Muñoz Ú, García-Magariño M, Sádaba MC, Castilla-Cortázar I (2016). Partial IGF-1 deficiency induces brain oxidative damage and edema, which are ameliorated by replacement therapy. BioFactors (Oxford, England).

[24] Labandeira-Garcia JL, Costa-Besada MA, Labandeira CM, Villar-Cheda B, Rodríguez-Perez AI (2017). Insulin-like growth factor-1 and neuroinflammation. Front. Aging Neurosci..

[25] Myhre CL, Thygesen C, Villadsen B, Vollerup J, Ilkjær L, Krohn KT, Grebing M, Zhao S, Khan AM, Dissing-Olesen L, Jensen MS, Babcock AA, Finsen B (2019). Microglia express insulin-like growth factor-1 in the hippocampus of aged APP(swe)/PS1(ΔE9) transgenic mice. Front. Cell. Neurosci..

[26] Jiang J, Lv X, Liang B, Jiang H (2017). Circulating TNF-α levels increased and correlated negatively with IGF-I in postoperative cognitive dysfunction. Neurol. Sci..

[27] Mohsenpour H, Pesce M, Patruno A, Bahrami A, Pour PM, Farzaei MH (2021). A review of plant extracts and plant-derived natural compounds in the prevention/treatment of neonatal hypoxic-ischemic brain injury. Int. J. Mol. Sci..

[28] Tao T, Liu M, Chen M, Luo Y, Wang C, Xu T, Jiang Y, Guo Y, Zhang JH (2020). Natural medicine in neuroprotection for ischemic stroke: Challenges and prospective. Pharmacol. Ther..

[29] Chen F, Bai N, Yue F, Hao Y, Wang H, He Y, Lu K (2023). Effects of oral β-caryophyllene (BCP) treatment on perioperative neurocognitive disorders: Attenuation of neuroinflammation associated with microglial activation and reinforcement of autophagy activity in aged mice. Brain Res..

[30] Gupta A, Singh AK, Kumar R, Jamieson S, Pandey AK, Bishayee A (2021). Neuroprotective potential of ellagic acid: A critical review. Adv. Nutr..

[31] Bidanchi RM, Lalrindika L, Khushboo M, Bhanushree B, Dinata R, Das M, Nisa N, Lalrinzuali S, Manikandan B, Saeed-Ahmed L, Sanjeev S, Murthy MK, Roy VK, Gurusubramanian G (2022). Antioxidative, anti-inflammatory and anti-apoptotic action of ellagic acid against lead acetate induced testicular and hepato-renal oxidative damages and pathophysiological changes in male Long Evans rats. Environ. Pollut..

[32] Farbood Y, Sarkaki A, Dianat M, Khodadadi A, Haddad MK, Mashhadizadeh S (2015). Ellagic acid prevents cognitive and hippocampal long-term potentiation deficits and brain inflammation in rat with traumatic brain injury. Life Sci..

[33] Kiasalari Z, Heydarifard R, Khalili M, Afshin-Majd S, Baluchnejadmojarad T, Zahedi E, Sanaierad A, Roghani M (2017). Ellagic acid ameliorates learning and memory deficits in a rat model of Alzheimer's disease: An exploration of underlying mechanisms. Psychopharmacology (Berl).

[34] Bains M, Kaur J, Akhtar A, Kuhad A, Sah SP (2022). Anti-inflammatory effects of ellagic acid and vanillic acid against quinolinic acid-induced rat model of Huntington's disease by targeting IKK-NF-κB pathway. Eur. J. Pharmacol..

[35] Rosczyk HA, Sparkman NL, Johnson RW (2008). Neuroinflammation and cognitive function in aged mice following minor surgery. Exp. Gerontol..

[36] Wang W, Chen C, Wang Q, Ma JG, Li YS, Guan Z, Wang R, Chen X (2023). Electroacupuncture pretreatment preserves telomerase reverse transcriptase function and alleviates postoperative cognitive dysfunction by suppressing oxidative stress and neuroinflammation in aged mice. CNS Neurosci. Ther..

[37] Xie X, Shen Z, Hu C, Zhang K, Guo M, Wang F, Qin K (2021). Dexmedetomidine ameliorates postoperative cognitive dysfunction in aged mice. Neurochem. Res..

[38] Lin SS, Hung CF, Ho CC, Liu YH, Ho HC, Chung JG (2000). Effects of ellagic acid by oral administration on N-acetylation and metabolism of 2-aminofluorene in rat brain tissues. Neurochem. Res..

[39] Sohrabi M, Floden AM, Manocha GD, Klug MG, Combs CK (2020). IGF-1R inhibitor ameliorates neuroinflammation in an Alzheimer's disease transgenic mouse model. Front. Cell. Neurosci..

[40] Ma Q (2013). Role of nrf2 in oxidative stress and toxicity. Annu. Rev. Pharmacol. Toxicol..

[41] Loboda A, Damulewicz M, Pyza E, Jozkowicz A, Dulak J (2016). Role of Nrf2/HO-1 system in development, oxidative stress response and diseases: An evolutionarily conserved mechanism. Cell. Mol. Life Sci. CMLS.

[42] Kavran JM, McCabe JM, Byrne PO, Connacher MK, Wang Z, Ramek A, Sarabipour S, Shan Y, Shaw DE, Hristova K, Cole PA, Leahy DJ (2014). How IGF-1 activates its receptor. Elife.

[43] Vasilcanu D, Weng WH, Girnita A, Lui WO, Vasilcanu R, Axelson M, Larsson O, Larsson C, Girnita L (2006). The insulin-like growth factor-1 receptor inhibitor PPP produces only very limited resistance in tumor cells exposed to long-term selection. Oncogene.

[44] Jiang G, Wang W, Cao Q, Gu J, Mi X, Wang K, Chen G, Wang X (2015). Insulin growth factor-1 (IGF-1) enhances hippocampal excitatory and seizure activity through IGF-1 receptor-mediated mechanisms in the epileptic brain. Clin. Sci. (Lond).

[45] Feng X, Valdearcos M, Uchida Y, Lutrin D, Maze M, Koliwad SK (2017). Microglia mediate postoperative hippocampal inflammation and cognitive decline in mice. JCI Insight.

[46] Arroba AI, Campos-Caro A, Aguilar-Diosdado M, Valverde M (2018). IGF-1, inflammation and retinal degeneration: A close network. Front. Aging Neurosci..

[47] Chen X, Le Y, Tang SQ, He WY, He J, Wang YH, Wang HB (2022). Painful diabetic neuropathy is associated with compromised microglial IGF-1 signaling which can be rescued by green tea polyphenol EGCG in mice. Oxid. Med. Cell Longev..

[48] Lipińska L, Klewicka E, Sójka M (2014). The structure, occurrence and biological activity of ellagitannins: A general review. Acta Sci. Pol. Technol. Aliment.

[49] Zuccari G, Baldassari S, Ailuno G, Turrini F, Alfei S, Caviglioli G (2020). Formulation strategies to improve oral bioavailability of ellagic acid. Appl. Sci..

[50] Ribeiro M, Rosenstock TR, Oliveira AM, Oliveira CR, Rego AC (2014). Insulin and IGF-1 improve mitochondrial function in a PI-3K/Akt-dependent manner and reduce mitochondrial generation of reactive oxygen species in Huntington's disease knock-in striatal cells. Free Rad. Biol. Med..

[51] Mahran YF (2020). New insights into the protection of growth hormone in cisplatin-induced nephrotoxicity: The impact of IGF-1 on the Keap1-Nrf2/HO-1 signaling. Life Sci..

[52] Rojanathammanee L, Puig KL, Combs CK (2013). Pomegranate polyphenols and extract inhibit nuclear factor of activated T-cell activity and microglial activation in vitro and in a transgenic mouse model of Alzheimer disease. J. Nutr..

[53] He XM, Zhou YZ, Sheng S, Li JJ, Wang GQ, Zhang F (2020). Ellagic acid protects dopamine neurons via inhibition of NLRP3 inflammasome activation in microglia. Oxid. Med. Cell Longev..

[54] Zhang Y, Taveggia C, Melendez-Vasquez C, Einheber S, Raine CS, Salzer JL, Brosnan CF, John GR (2006). Interleukin-11 potentiates oligodendrocyte survival and maturation, and myelin formation. J. Neurosci..

[55] Tang Y, Le W (2016). Differential roles of M1 and M2 microglia in neurodegenerative diseases. Mol. Neurobiol..

[56] Orihuela R, McPherson CA, Harry GJ (2016). Microglial M1/M2 polarization and metabolic states. Br. J. Pharmacol..

[57] Yao K, Zhao YF (2018). Aging modulates microglia phenotypes in neuroinflammation of MPTP-PD mice. Exp. Gerontol..

[58] Liu YL, Huang HJ, Sheu SY, Liu YC, Lee IJ, Chiang SC, Lin AM (2023). Oral ellagic acid attenuated LPS-induced neuroinflammation in rat brain: MEK1 interaction and M2 microglial polarization. Exp. Biol. Med..

[59] Pristerà A, Blomeley C, Lopes E, Threlfell S, Merlini E, Burdakov D, Cragg S, Guillemot F, Ang SL (2019). Dopamine neuron-derived IGF-1 controls dopamine neuron firing, skill learning, and exploration. Proc. Natl. Acad. Sci. USA.

[60] Chen X, Le Y, He WY, He J, Wang YH, Zhang L, Xiong QM, Zheng XQ, Liu KX, Wang HB (2021). Abnormal insulin-like growth factor 1 signaling regulates neuropathic pain by mediating the mechanistic target of rapamycin-related autophagy and neuroinflammation in mice. ACS Chem. Neurosci..

[61] Li X, Yu W, Guan Y, Zou H, Liang Z, Huang M, Zhao R, Zhao C, Ren Z, Chen Z (2020). Peripheral circulation and astrocytes contribute to the MSC-mediated increase in IGF-1 levels in the infarct cortex in a dMCAO rat model. Stem Cells Int..

[62] Ivan DC, Berve KC, Walthert S, Monaco G, Borst K, Bouillet E, Ferreira F, Lee H, Steudler J, Buch T, Prinz M, Engelhardt B, Locatelli G (2023). Insulin-like growth factor-1 receptor controls the function of CNS-resident macrophages and their contribution to neuroinflammation. Acta Neuropathol. Commun..

[63] Arjunan A, Sah DK, Woo M, Song J (2023). Identification of the molecular mechanism of insulin-like growth factor-1 (IGF-1): A promising therapeutic target for neurodegenerative diseases associated with metabolic syndrome. Cell Biosci..

[64] Sun Z, Wu K, Gu L, Huang L, Zhuge Q, Yang S, Wang Z (2020). IGF-1R stimulation alters microglial polarization via TLR4/NF-κB pathway after cerebral hemorrhage in mice. Brain Res. Bull..

[65] Wan Y, Gao W, Zhou K, Liu X, Jiang W, Xue R, Wu W (2022). Role of IGF-1 in neuroinflammation and cognition deficits induced by sleep deprivation. Neurosci. Lett..

[66] Sonntag WE, Ramsey M, Carter CS (2005). Growth hormone and insulin-like growth factor-1 (IGF-1) and their influence on cognitive aging. Ageing Res. Rev..

[67] Jung HJ, Suh Y (2014). Regulation of IGF -1 signaling by microRNAs. Front. Genet..

[68] Bai S, Yu Y, An L, Wang W, Fu X, Chen J, Ma J (2022). Ellagic acid increases stress resistance via insulin/IGF-1 signaling pathway in Caenorhabditis elegans. Molecules.

[69] Jurga AM, Paleczna M, Kuter KZ (2020). Overview of general and discriminating markers of differential microglia phenotypes. Front. Cell. Neurosci..

