# Supplementary material for: Oral administration of ellagic acid mitigates perioperative neurocognitive disorders, hippocampal oxidative stress, and neuroinflammation in aged mice by restoring IGF-1 signaling
Source: Sci Rep. 2024 Jan 30;14:2509. doi: 10.1038/s41598-024-53127-8 (PMC10827749; doi:10.1038/s41598-024-53127-8)

Figure 1B-IGF-1

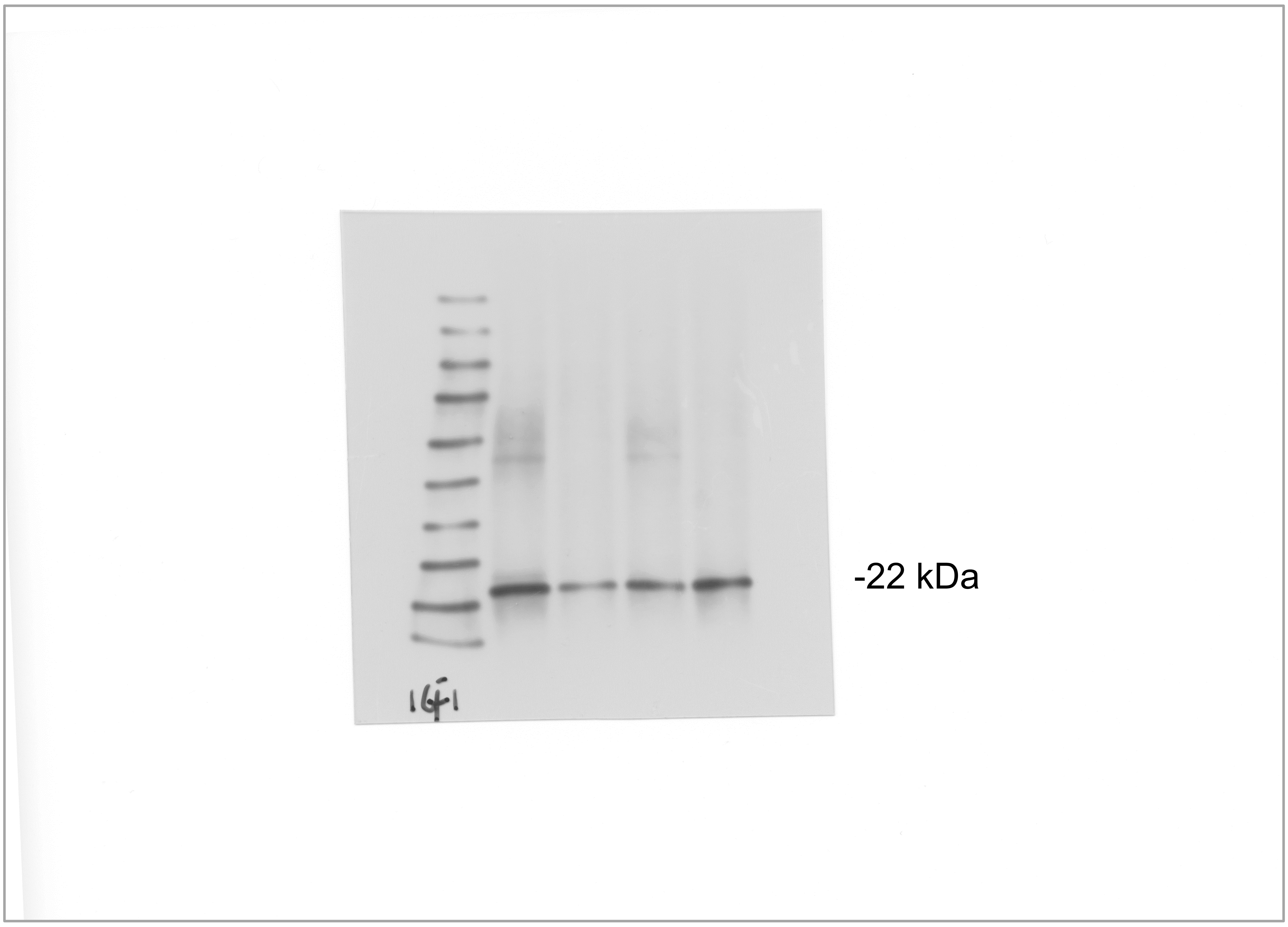

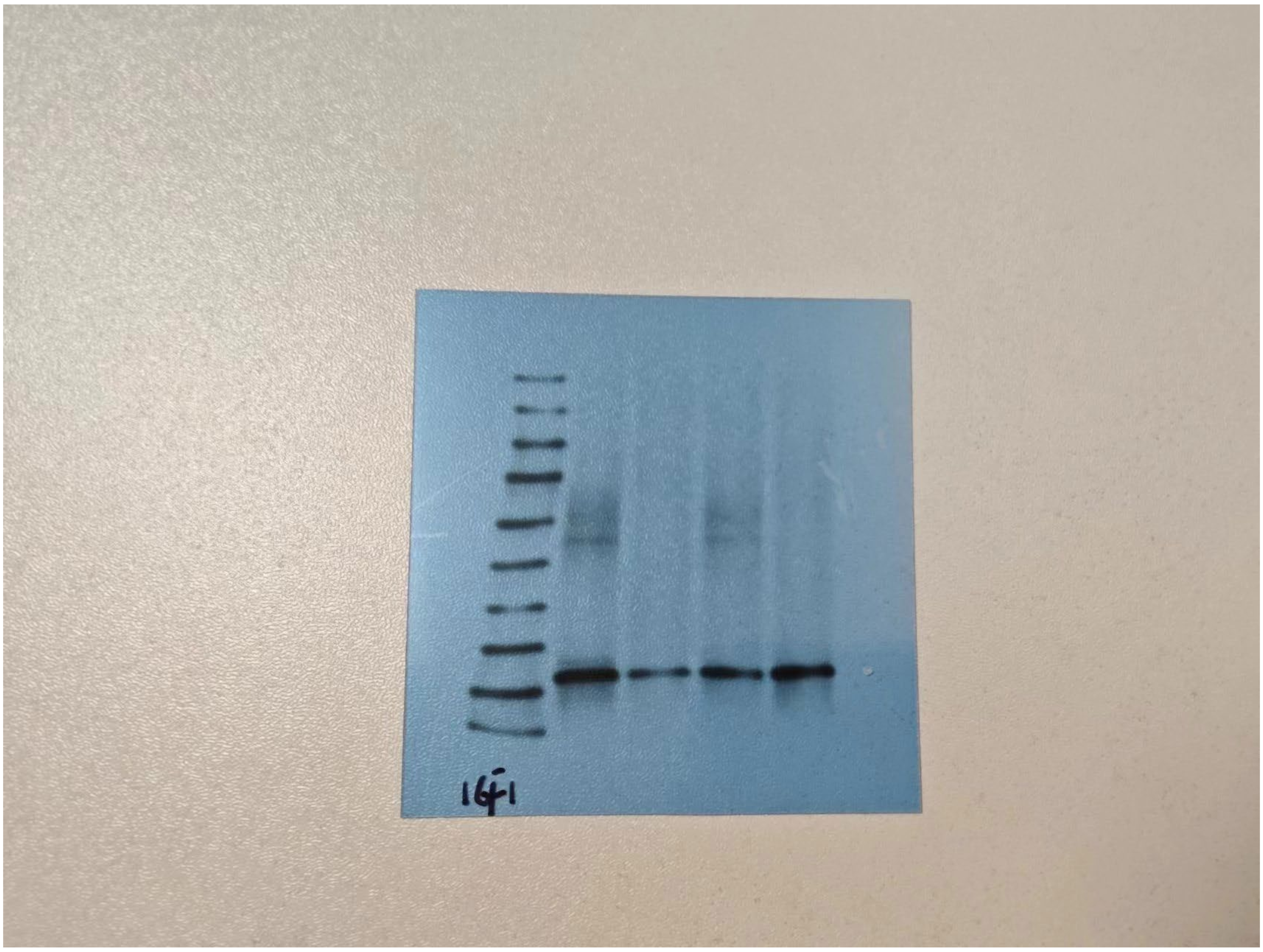

Figure 1B- β-actin

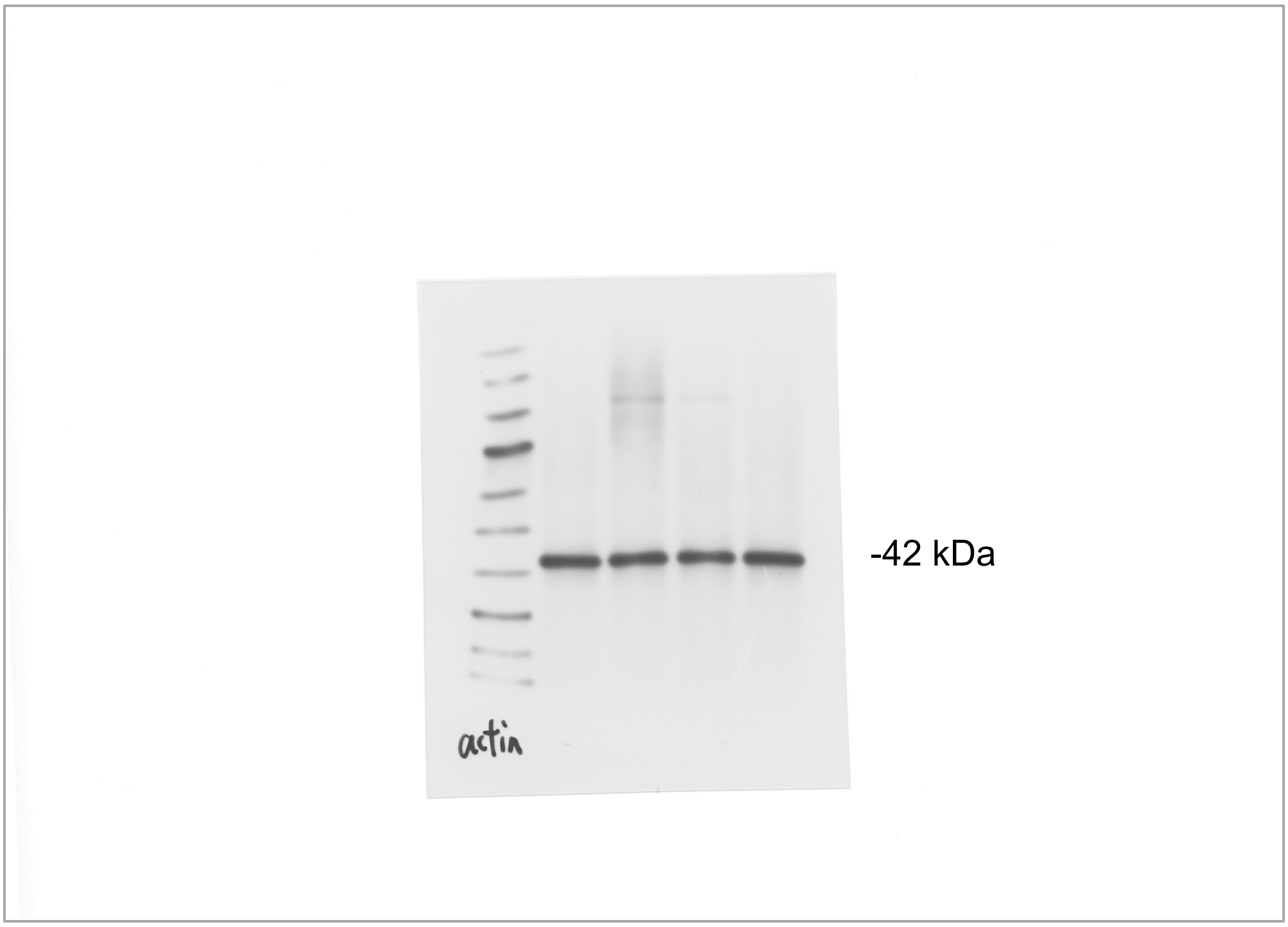

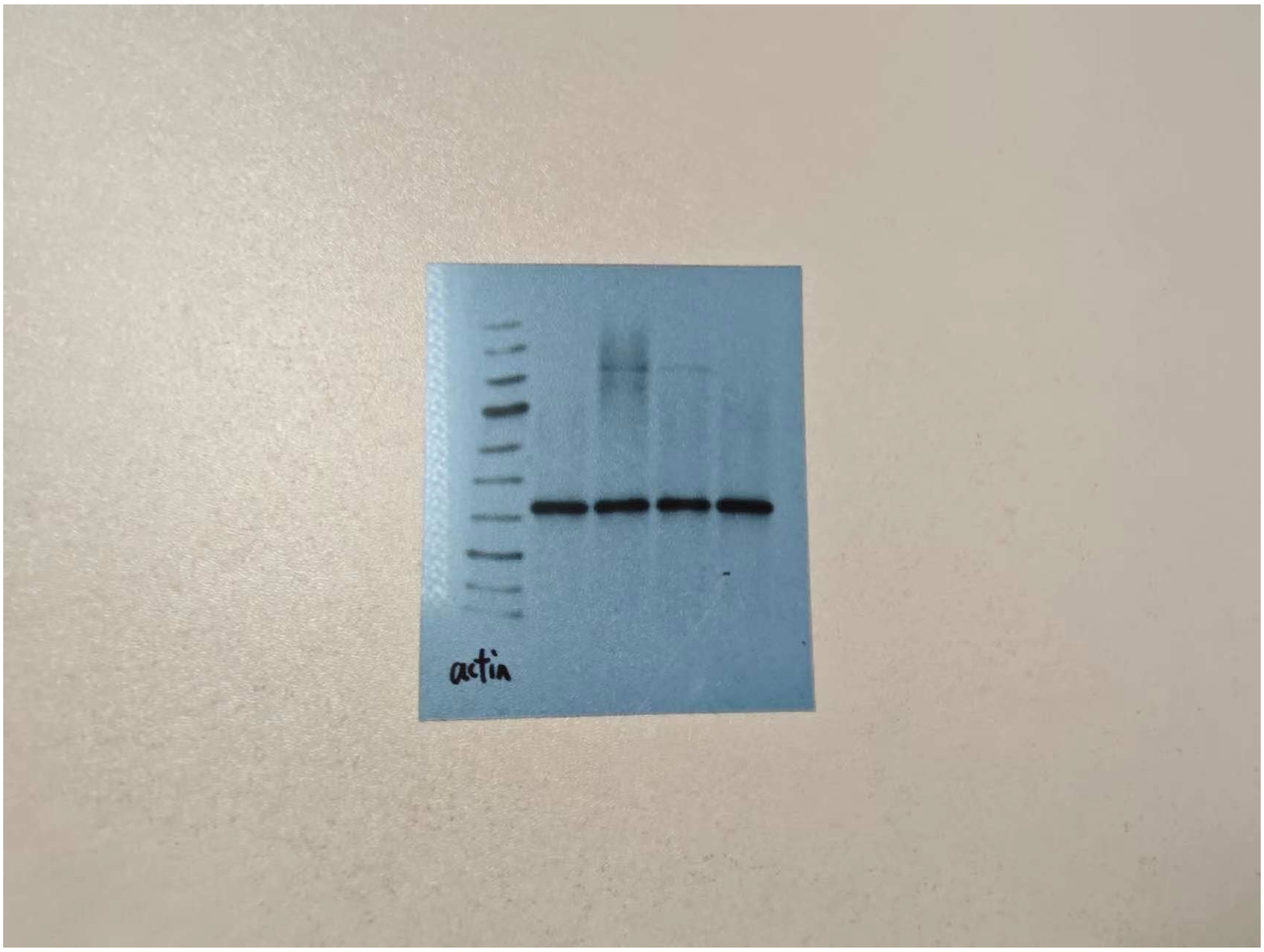

Figure 1C-IGF-1

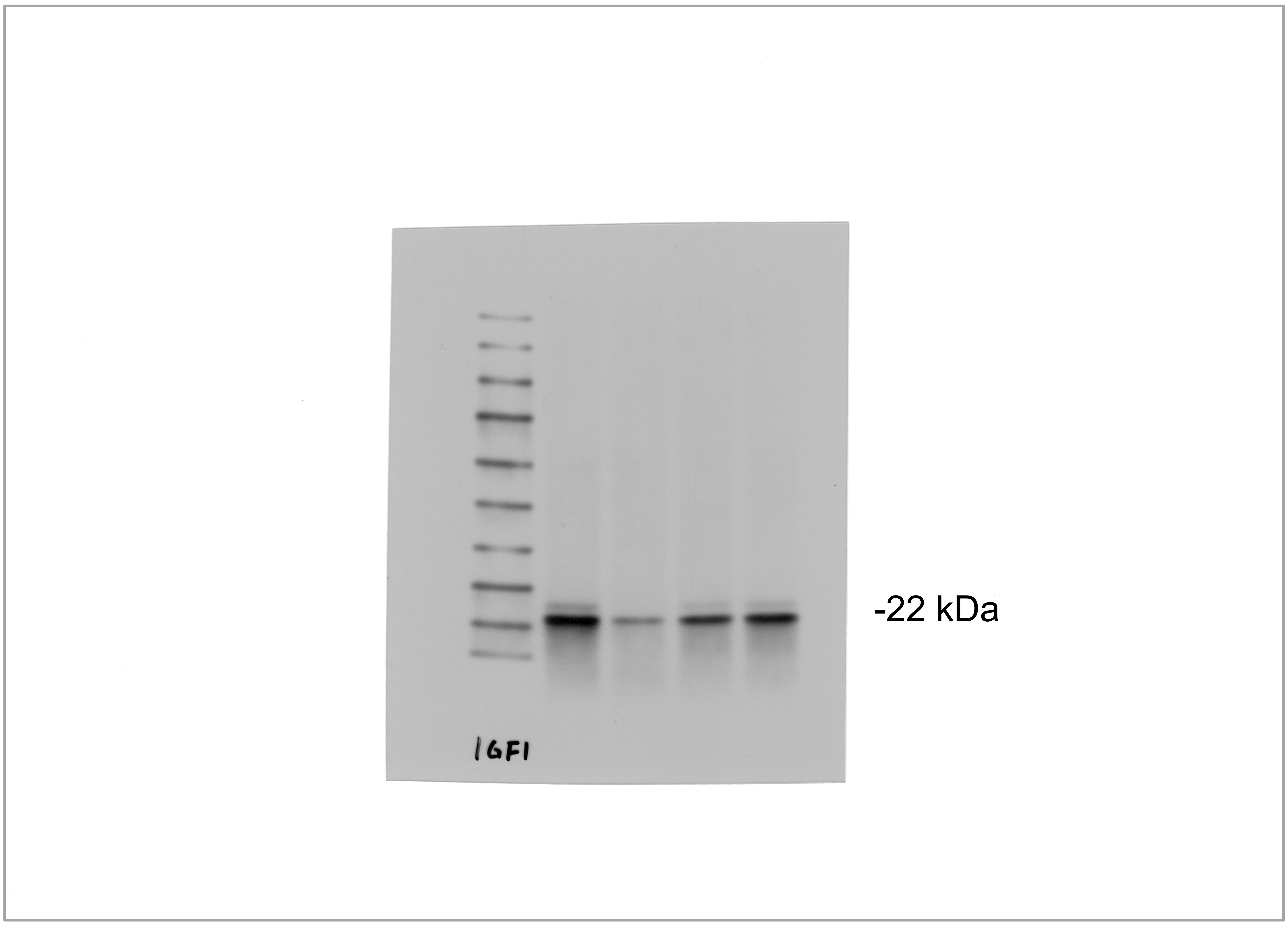

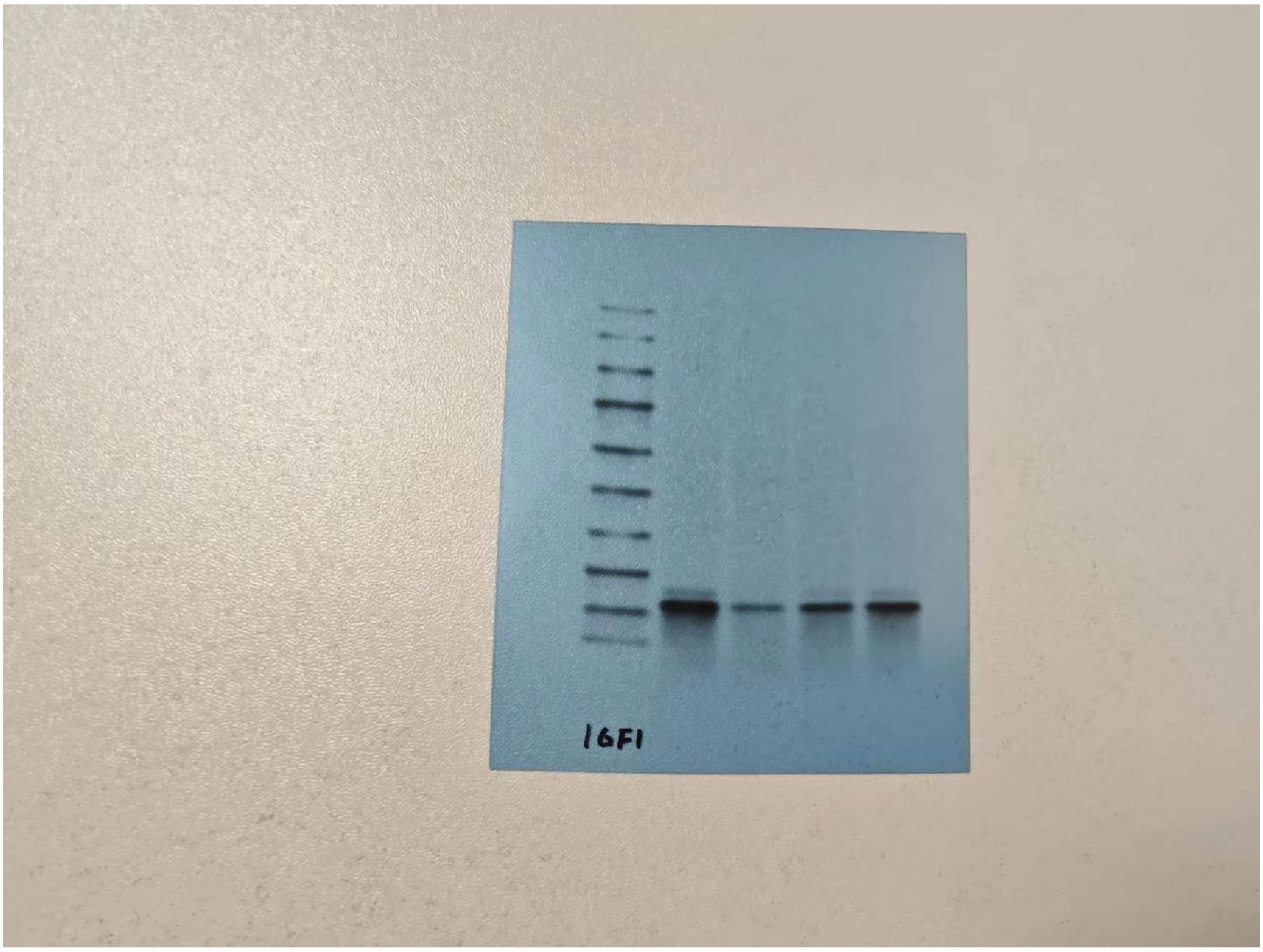

Figure 1C- p-IGF1R

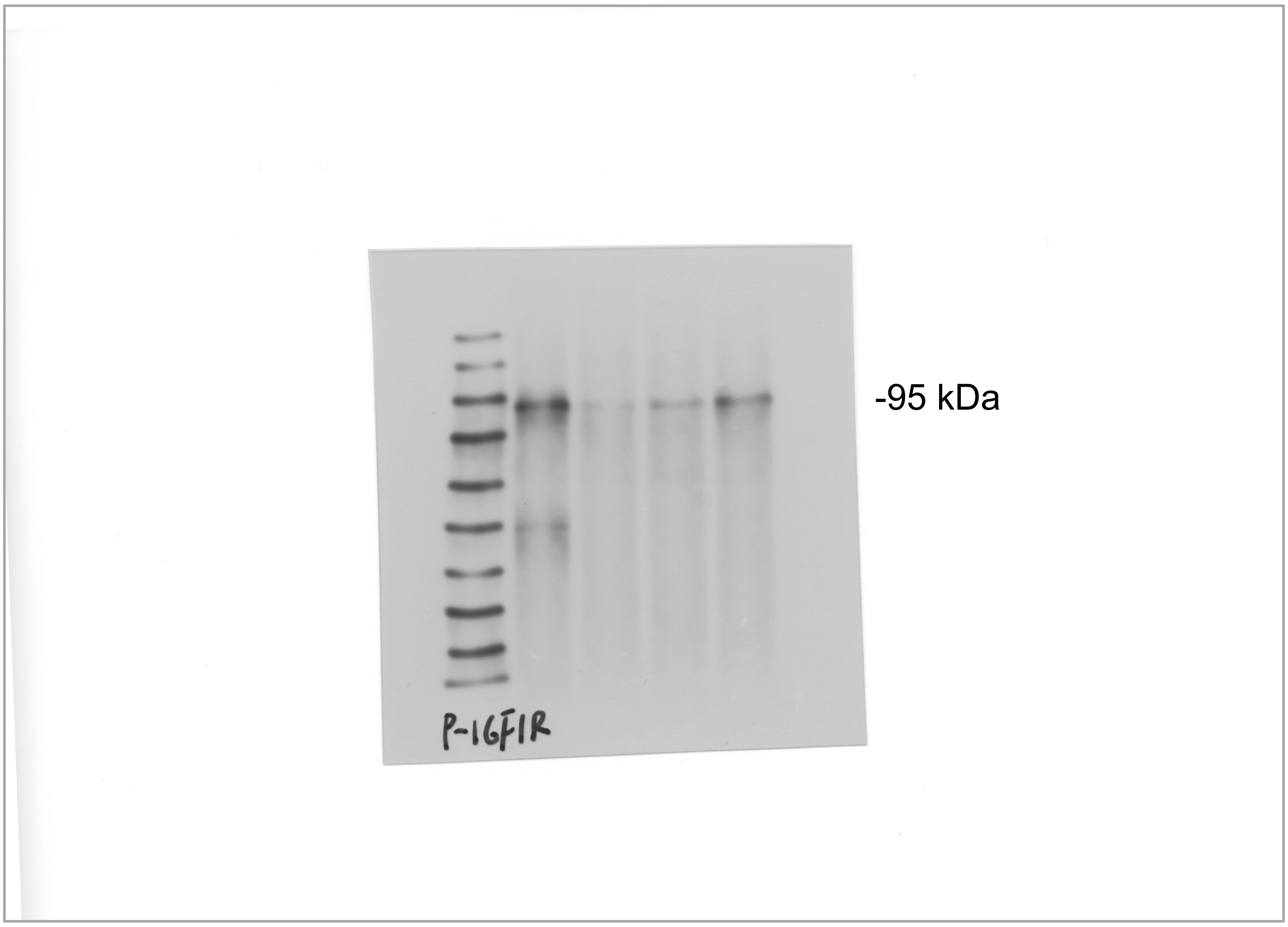

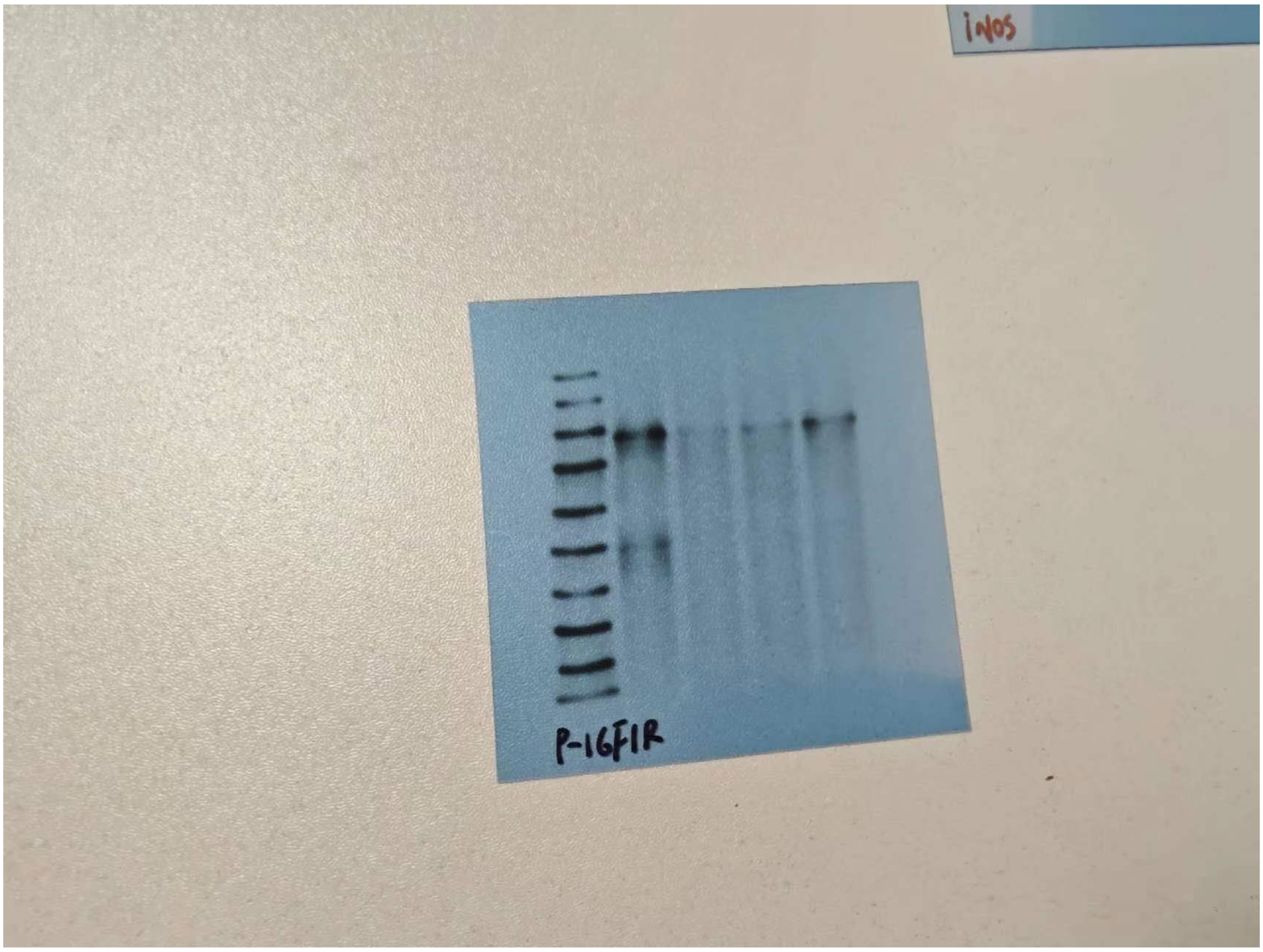

Figure 1C- IGF1R

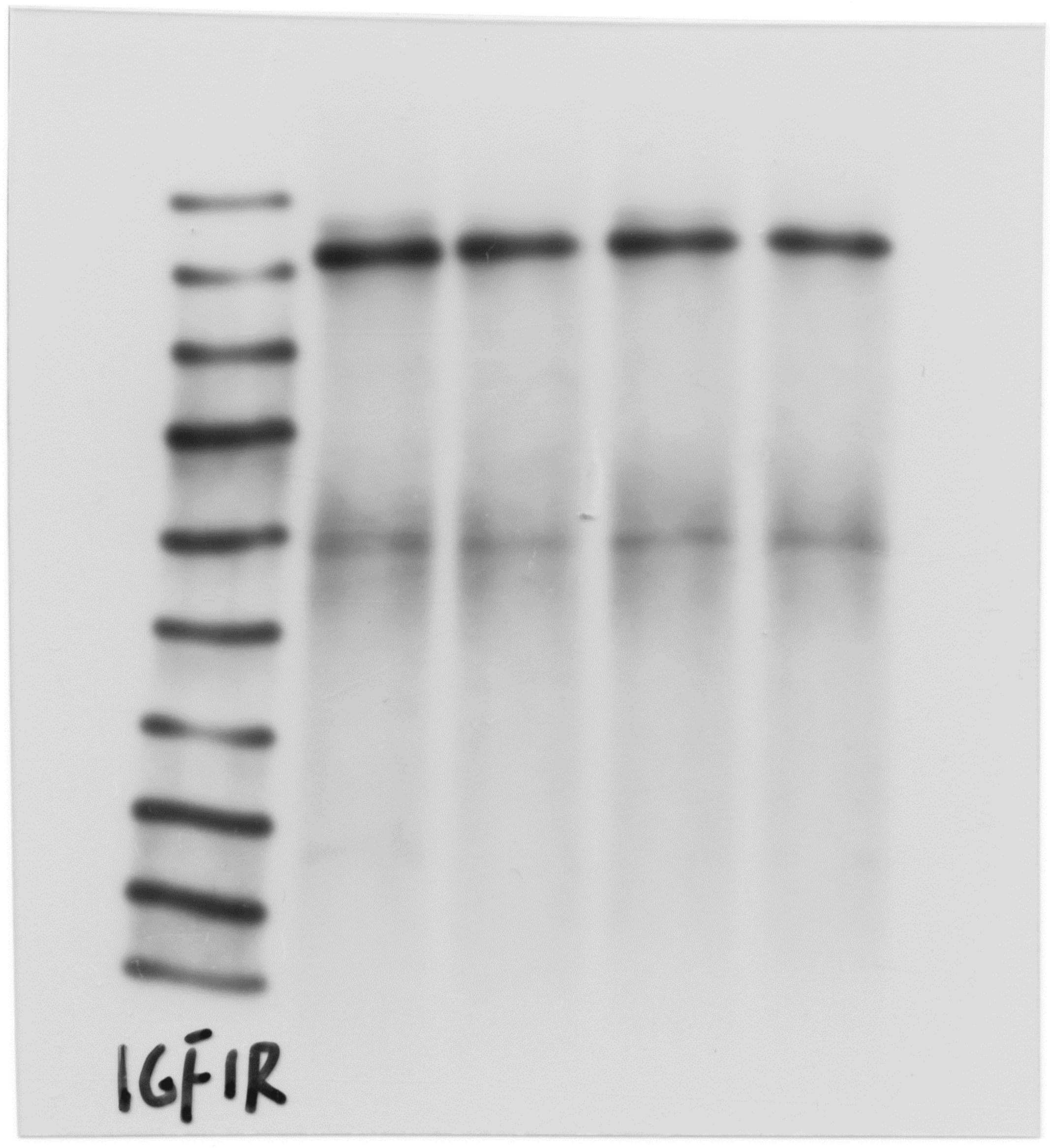

-95 kDa

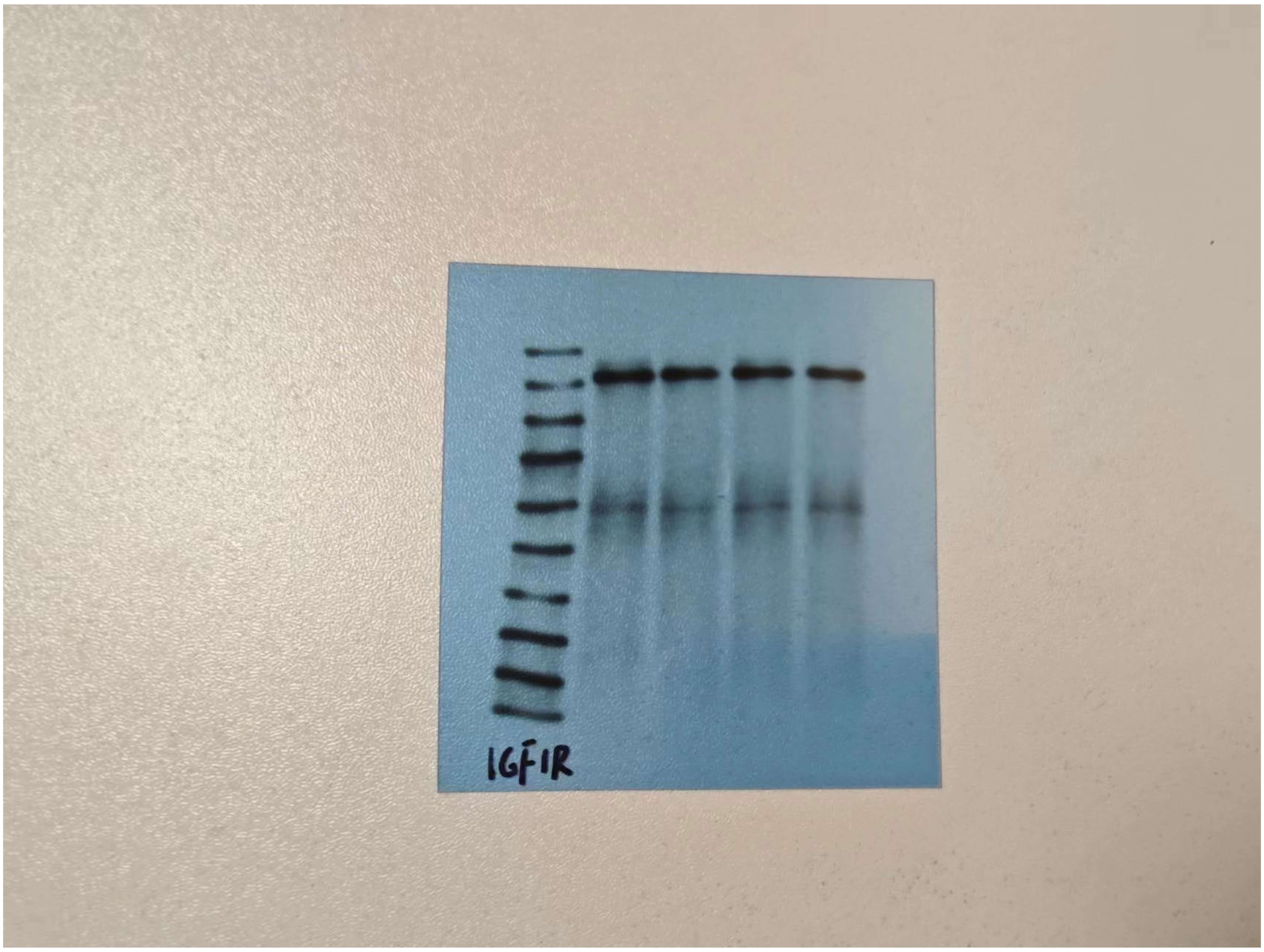

Figure 1C- β-actin

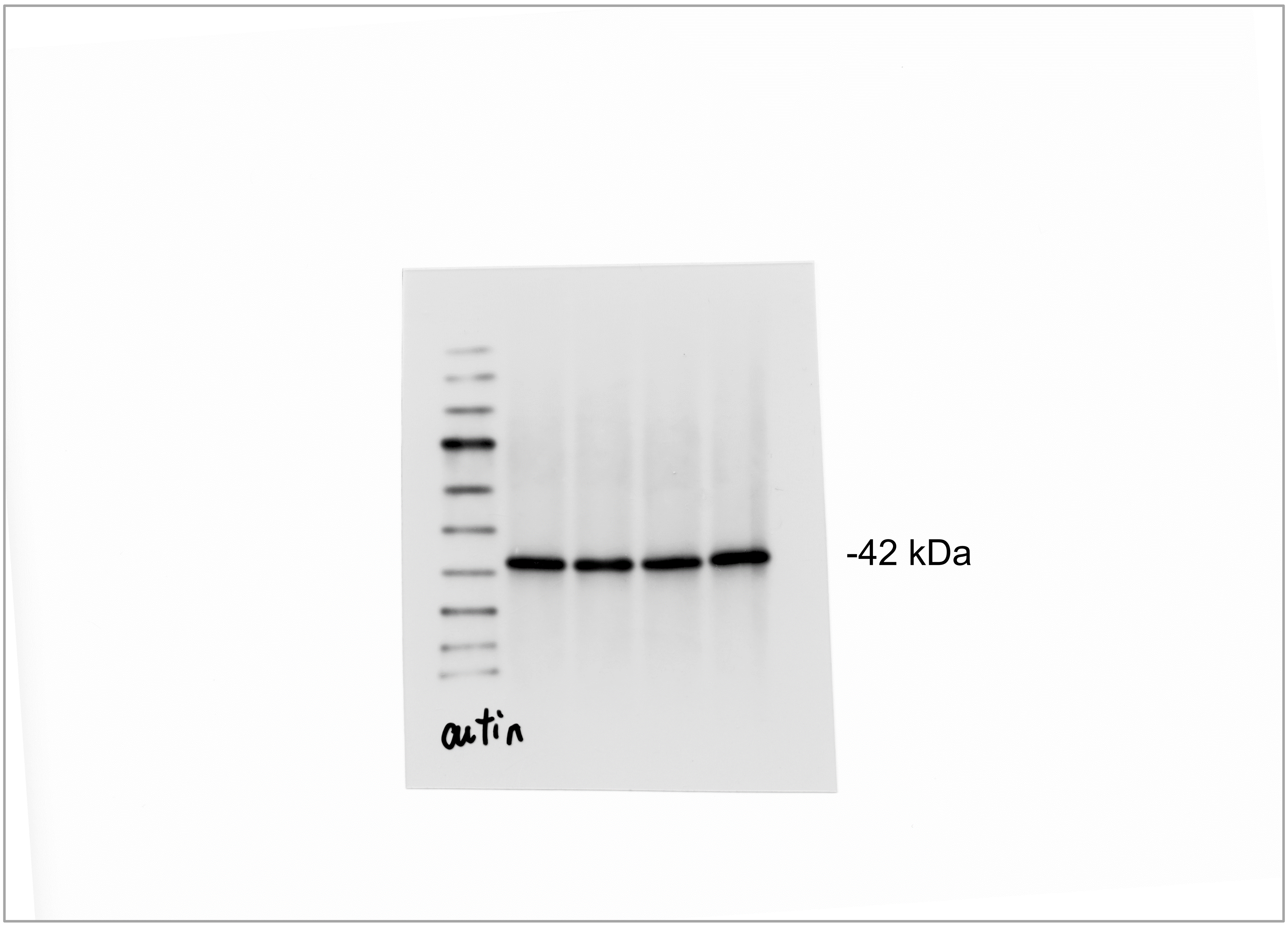

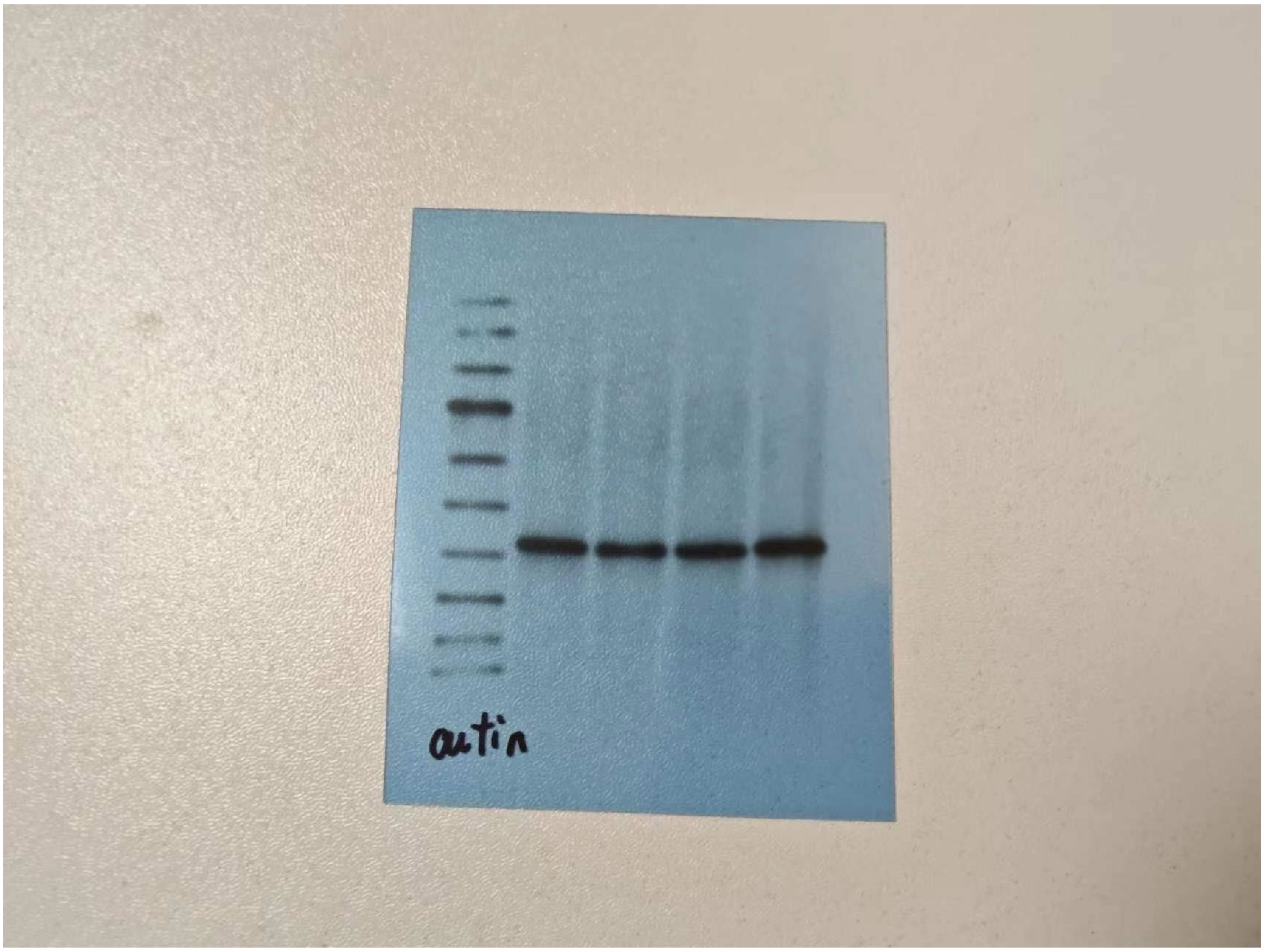

Figure 3E- Nrf2

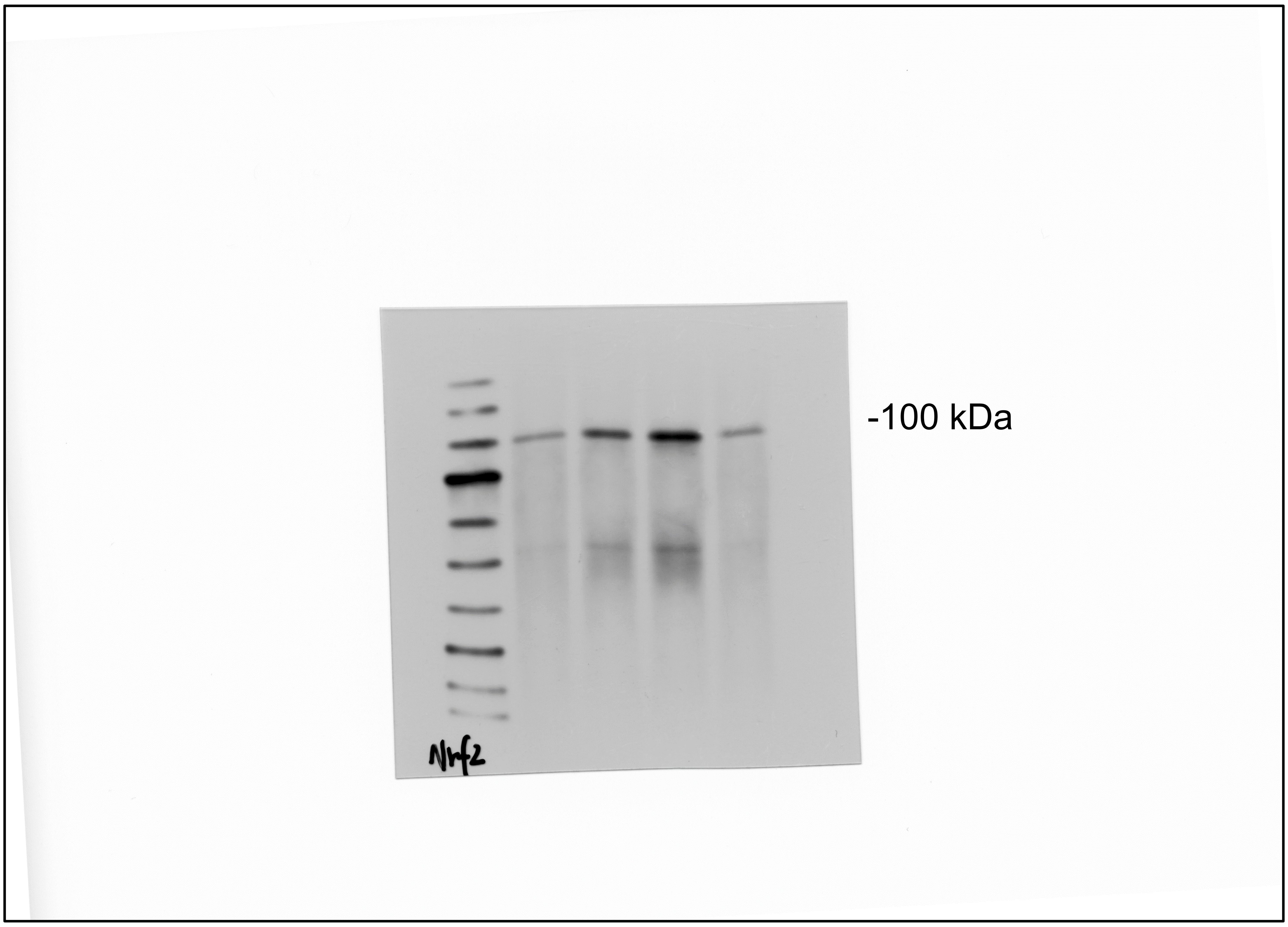

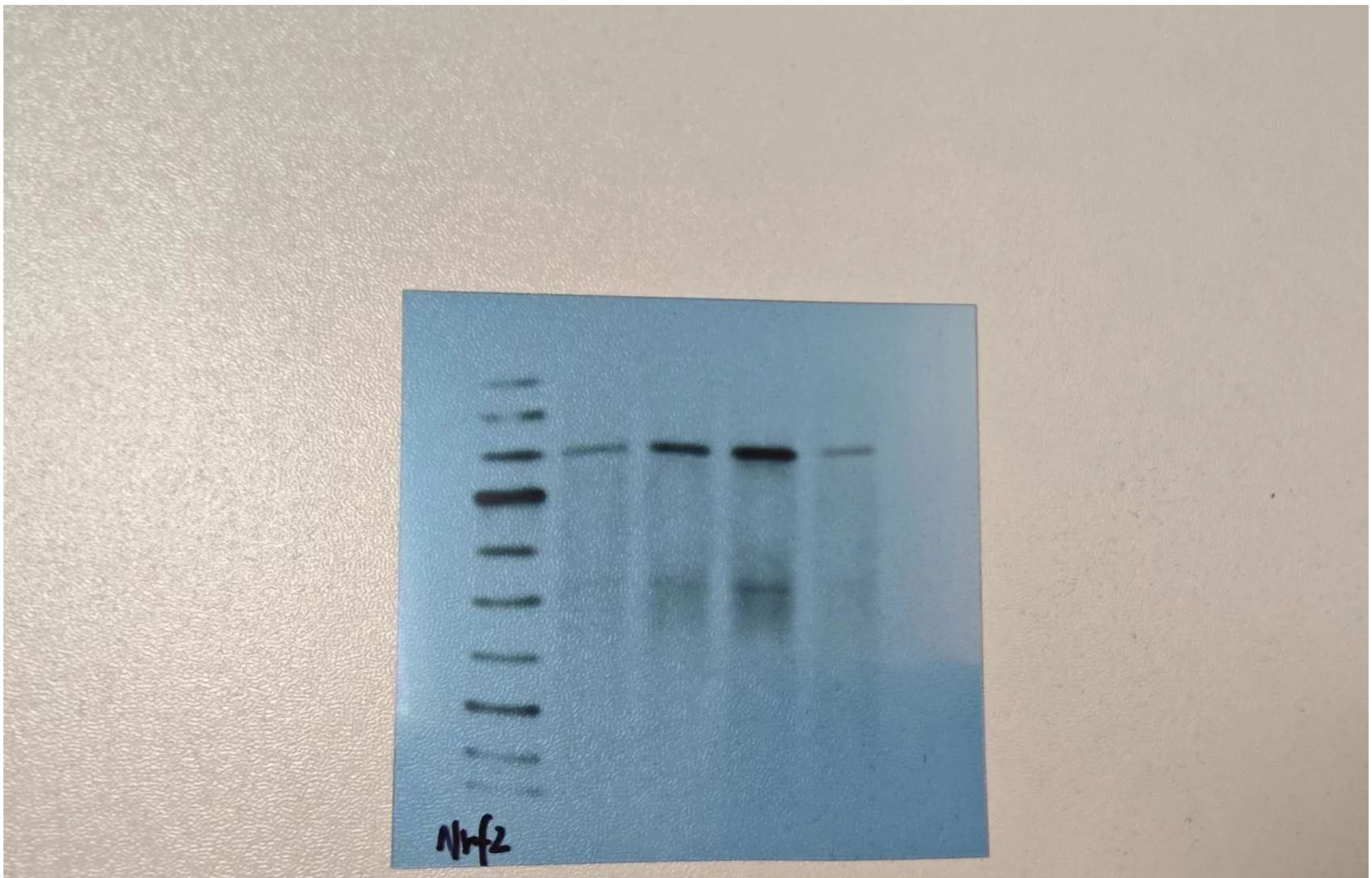

Figure 3E- HO-1

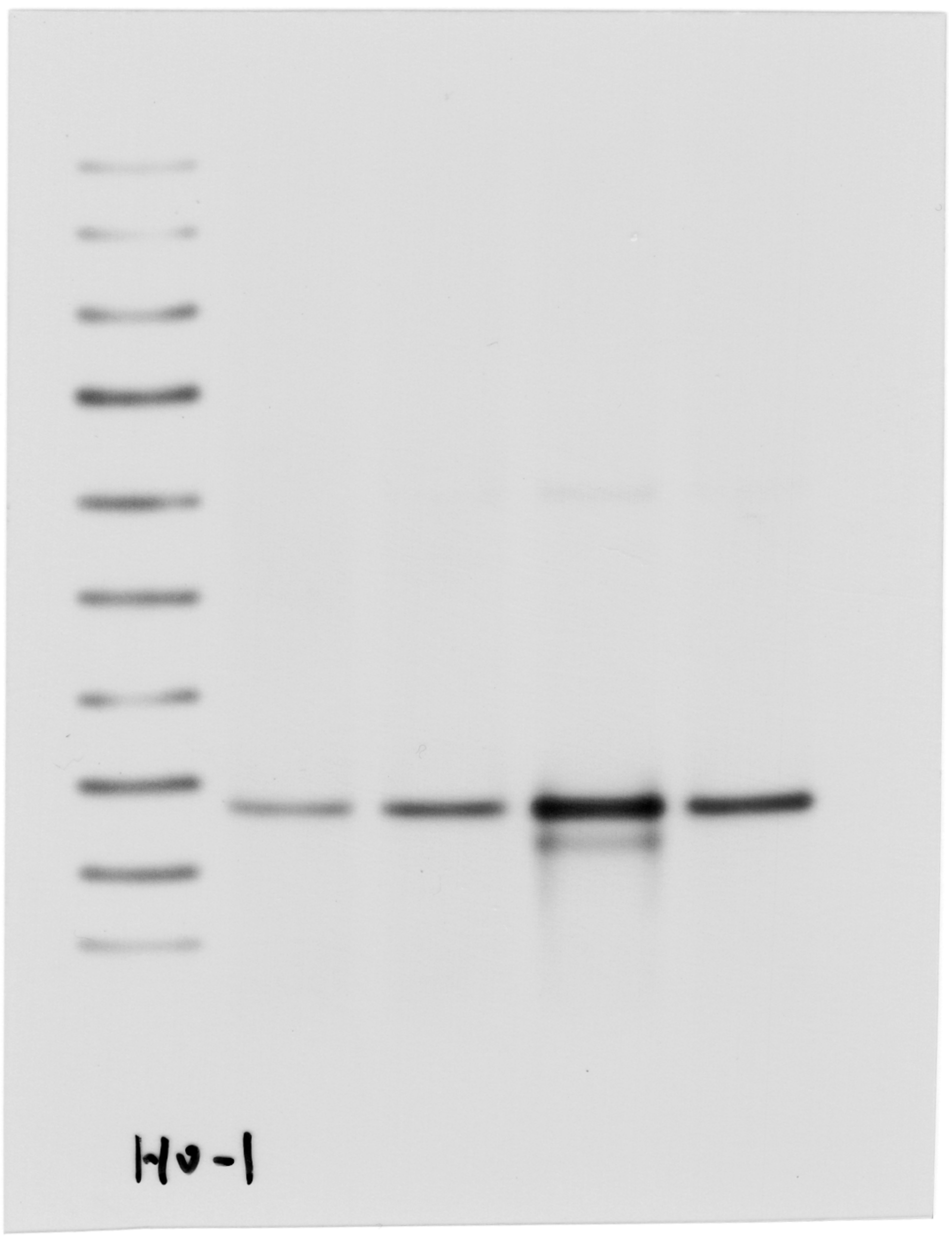

-28 kDa

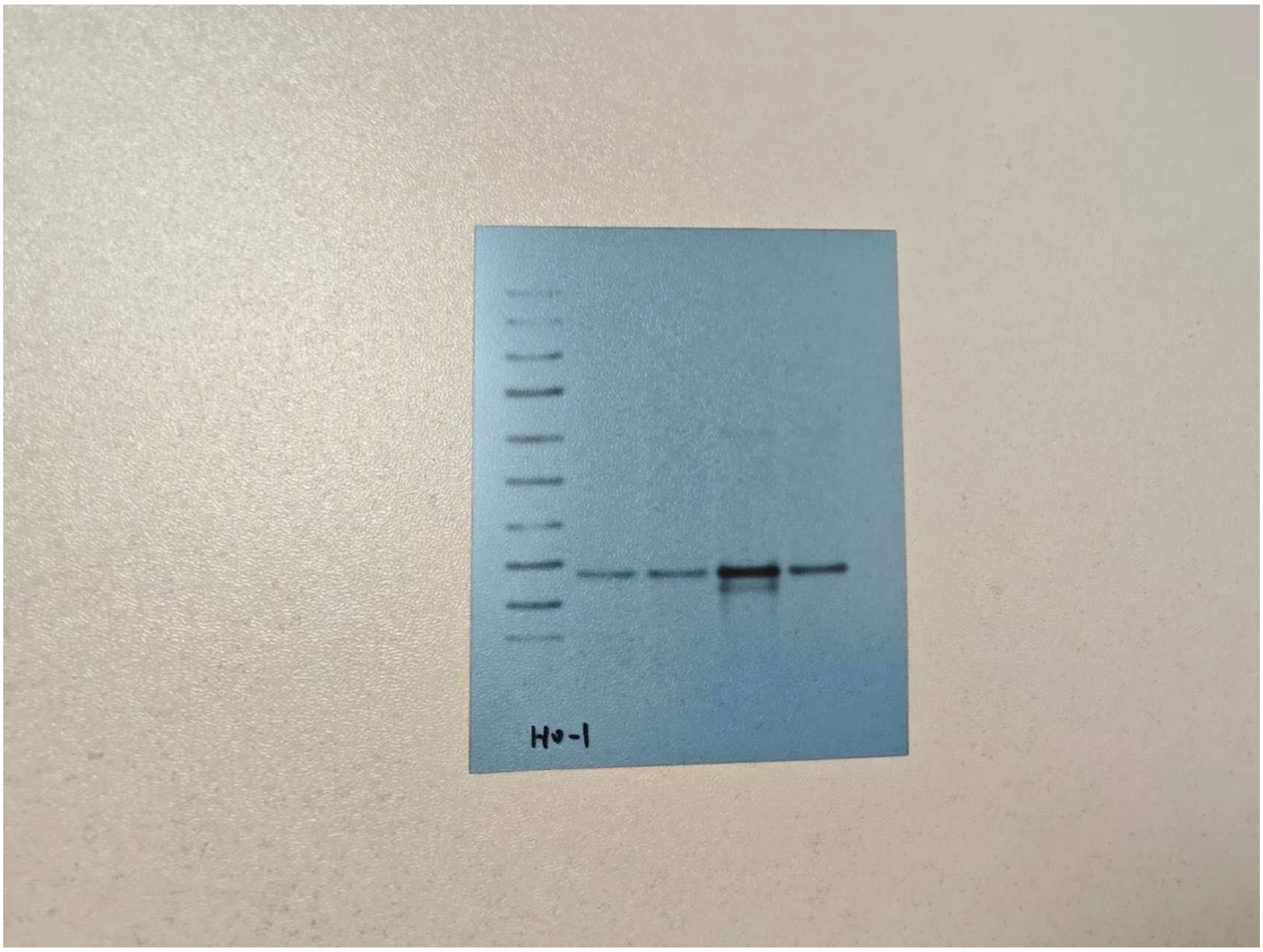

Figure 3E- LaminB

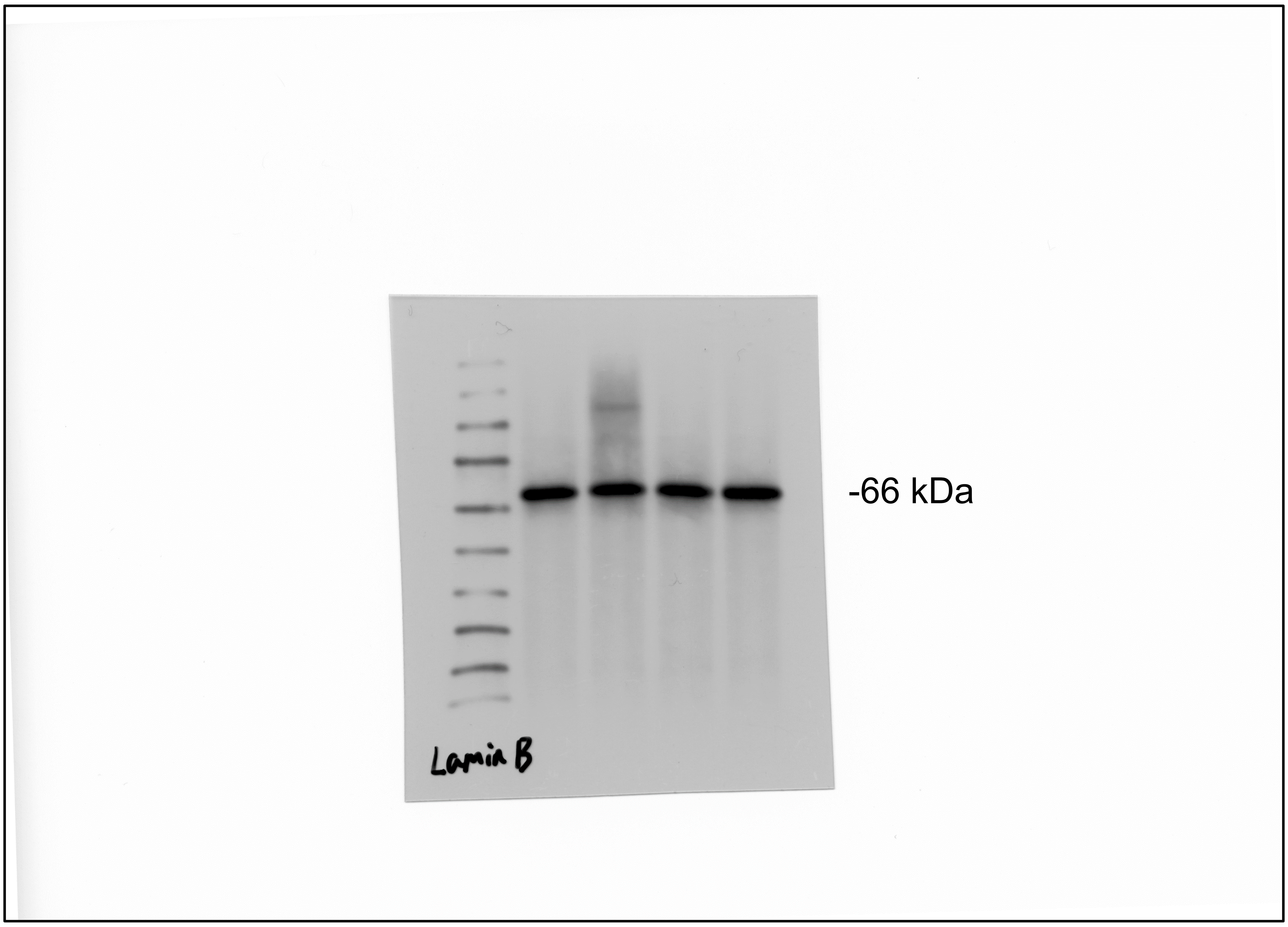

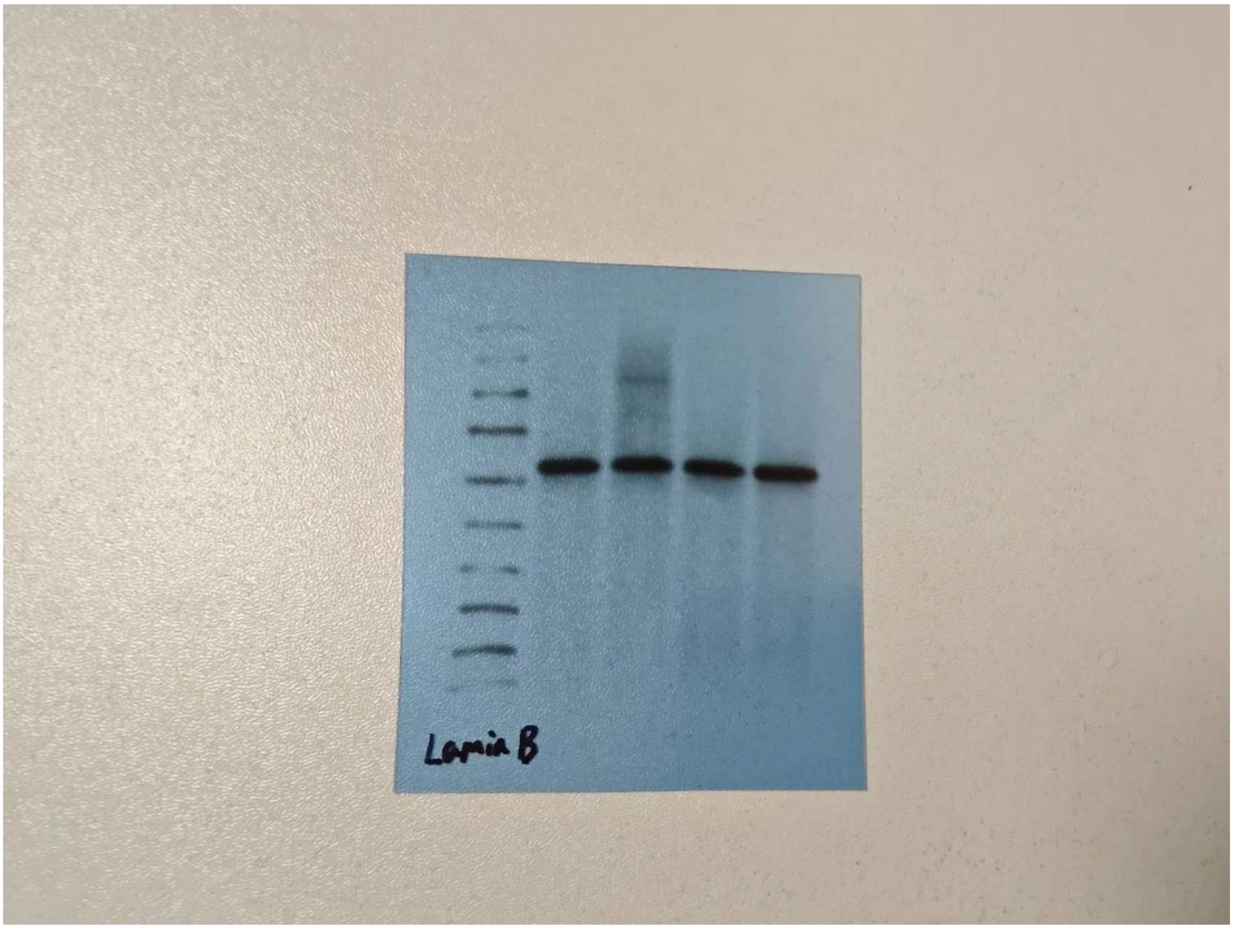

Figure 5G- iNOS

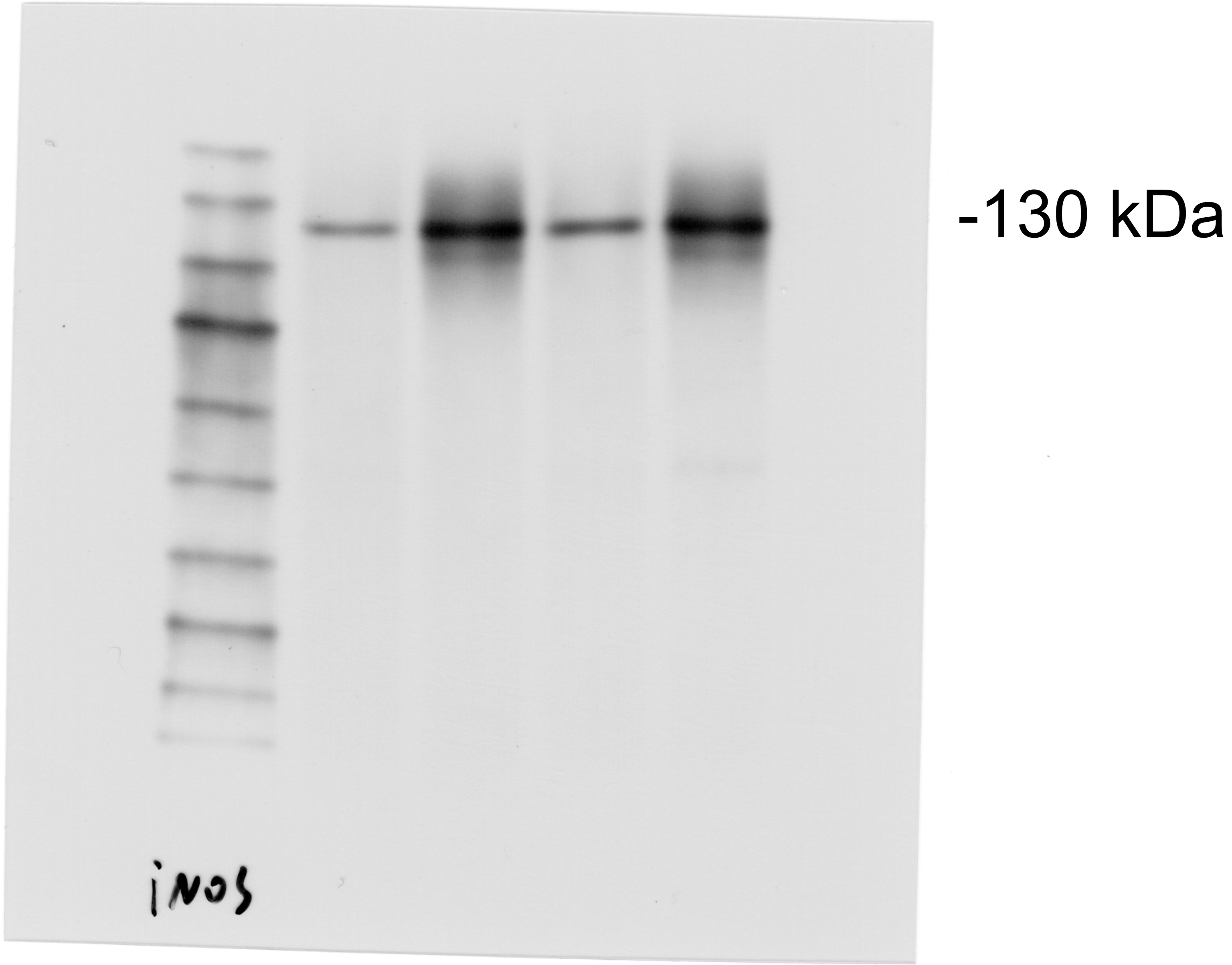

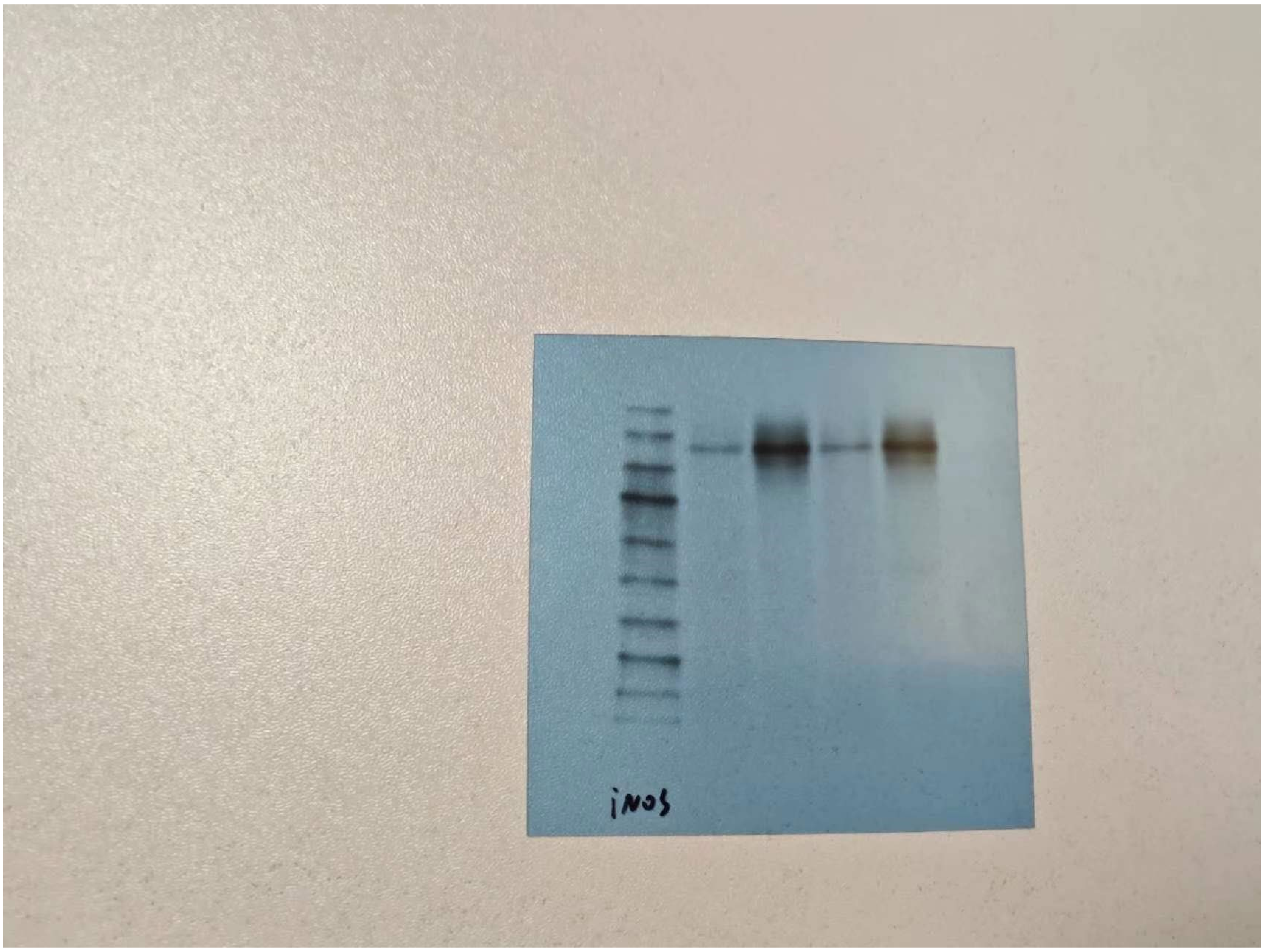

Figure 5G- Arg-1

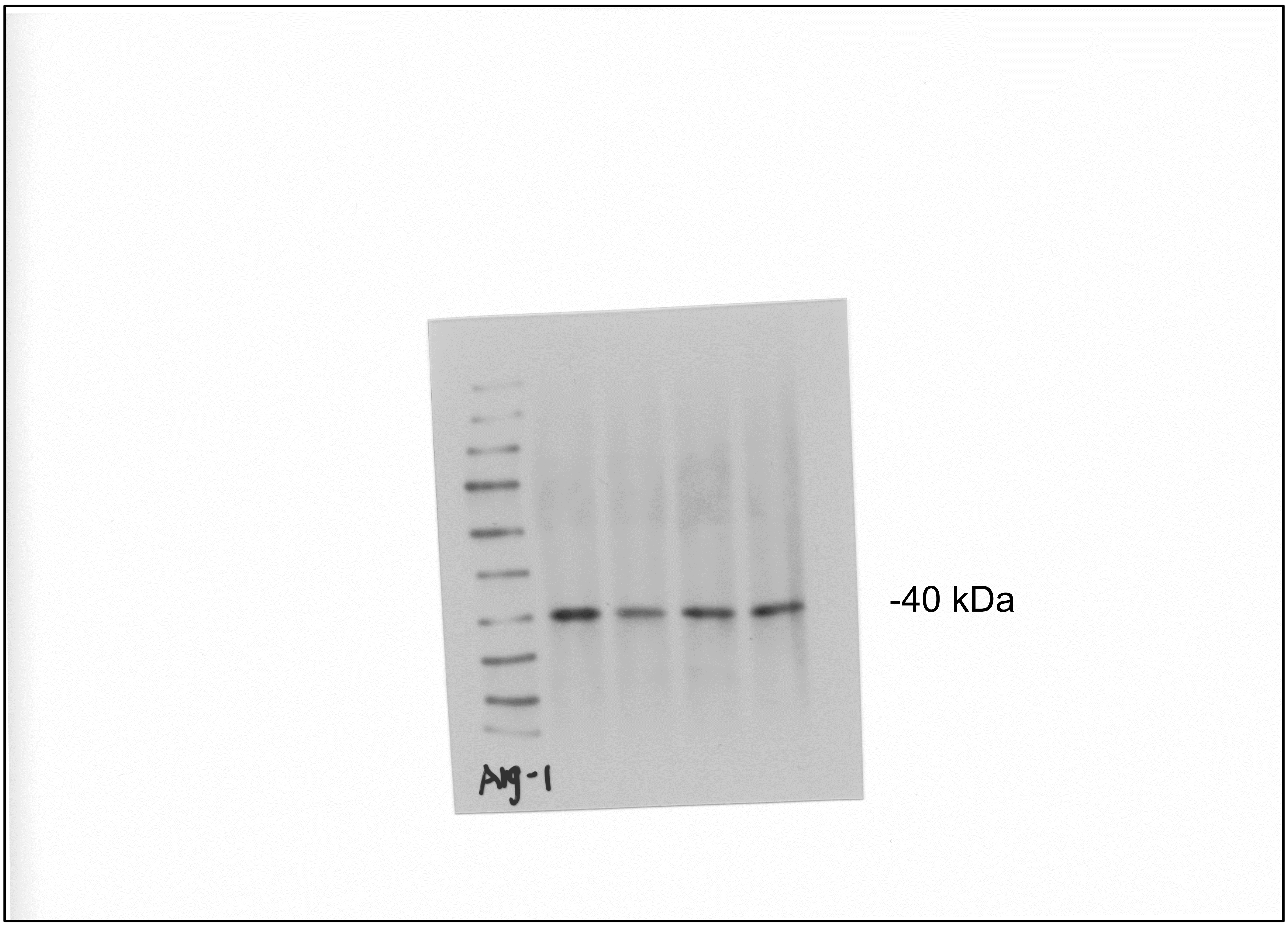

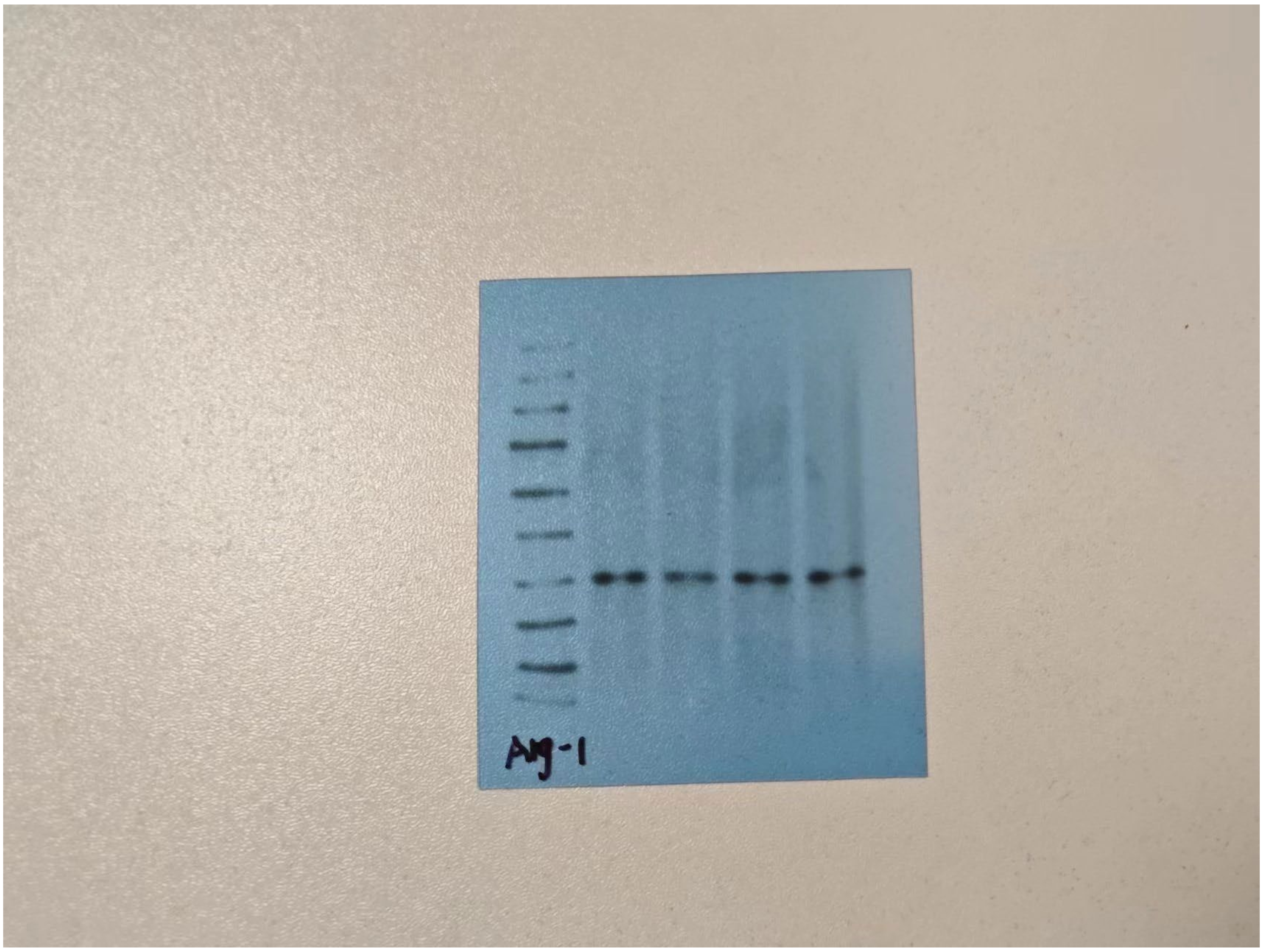

Figure 5G- β-actin

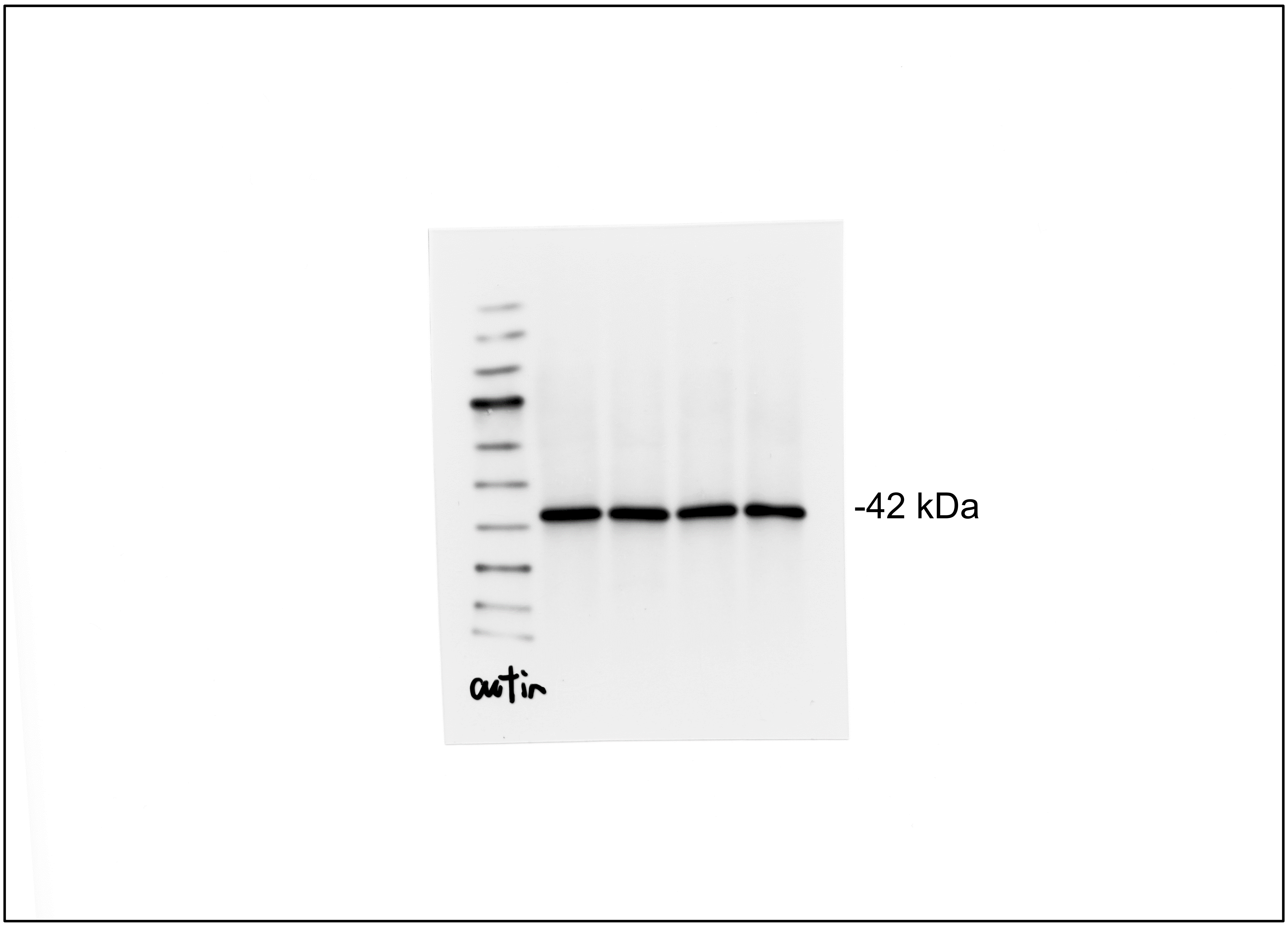

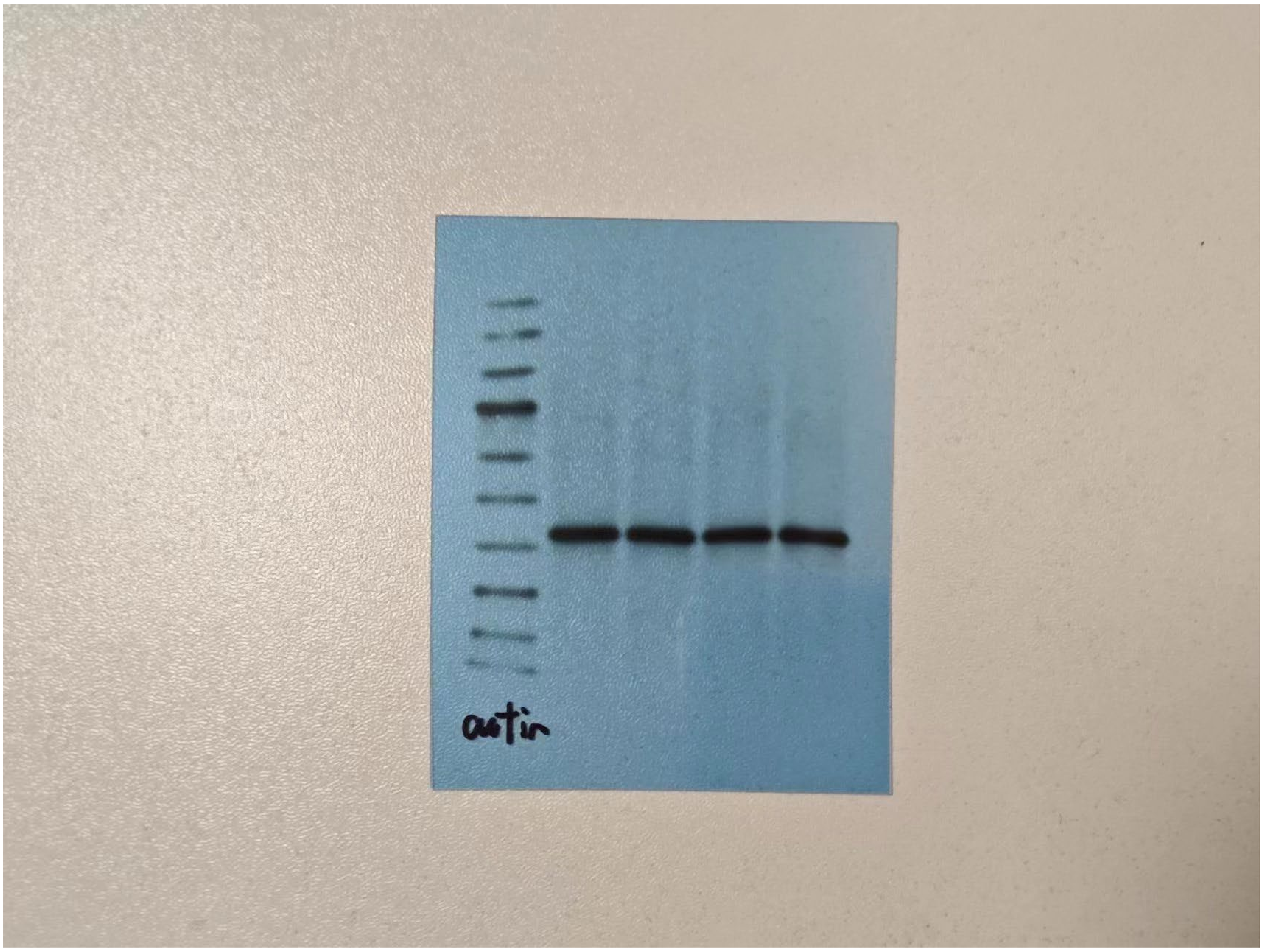

All Films Collection

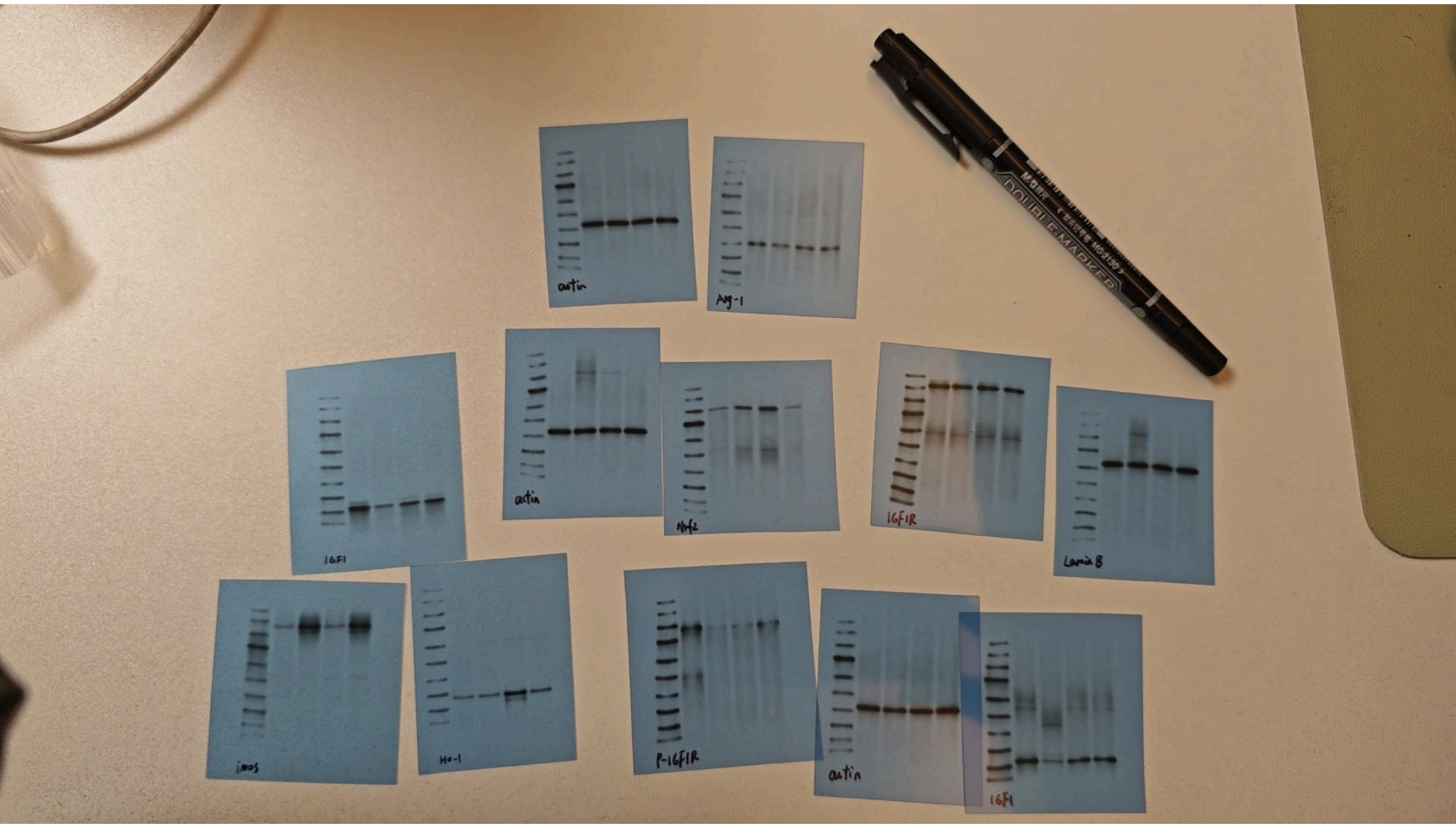

Supplement: Supplementary file 1 — Supplementary Figures. [file 41598_2024_53127_MOESM1_ESM.pdf]
